# Development of a Bioinformatics Framework for the Detection of Gene Conversion and the Analysis of Combinatorial Diversity in Immunoglobulin Heavy Chains in Four Cattle Breeds

**DOI:** 10.1371/journal.pone.0164567

**Published:** 2016-11-09

**Authors:** Stefanie Walther, Manfred Tietze, Claus-Peter Czerny, Sven König, Ulrike S. Diesterbeck

**Affiliations:** 1 Department of Animal Sciences, Institute of Veterinary Medicine, Division of Microbiology and Animal Hygiene, Faculty of Agricultural Sciences, Georg-August University Goettingen, Goettingen, Germany; 2 Department of Animal Breeding, University of Kassel, Witzenhausen, Germany; Qom University, ISLAMIC REPUBLIC OF IRAN

## Abstract

We have developed a new bioinformatics framework for the analysis of rearranged bovine heavy chain immunoglobulin (Ig) variable regions by combining and refining widely used alignment algorithms. This bioinformatics framework allowed us to investigate alignments of heavy chain framework regions (FRHs) and the separate alignments of FRHs and heavy chain complementarity determining regions (CDRHs) to determine their germline origin in the four cattle breeds Aubrac, German Black Pied, German Simmental, and Holstein Friesian. Now it is also possible to specifically analyze Ig heavy chains possessing exceptionally long CDR3Hs. In order to gain more insight into breed specific differences in Ig combinatorial diversity, somatic hypermutations and putative gene conversions of IgG, we compared the dominantly transcribed variable (IGHV), diversity (IGHD), and joining (IGHJ) segments and their recombination in the four cattle breeds. The analysis revealed the use of 15 different *IGHV* segments, 21 *IGHD* segments, and two *IGHJ* segments with significant different transcription levels within the breeds. Furthermore, there are preferred rearrangements within the three groups of CDR3H lengths. In the sequences of group 2 (CDR3H lengths (L) of 11–47 amino acid residues (aa)) a higher number of recombination was observed than in sequences of group 1 (L≤10 aa) and 3 (L≥48 aa). The combinatorial diversity of germline *IGHV*, *IGHD*, and *IGHJ*-segments revealed 162 rearrangements that were significantly different. The few preferably rearranged gene segments within group 3 CDR3H regions may indicate specialized antibodies because this length is unique in cattle. The most important finding of this study, which was enabled by using the bioinformatics framework, is the discovery of strong evidence for gene conversion as a rare event using pseudogenes fulfilling all definitions for this particular diversification mechanism.

## Introduction

The basic genetic mechanism in developing immunoglobulin diversity is similar in all jawed vertebrates. Immunoglobulins (Ig) are Y-shaped hetero-tetramers consisting of two identical heavy chains (IGH) and two identical light chains, either κ or λ in mammals (IGK, IGL) [1]. Both chains are functionally divided into variable and constant domains that are combined during B-cell development. The variable domain is rearranged by separate heavy and light chain variable (*IGHV*, *IGKV*, *IGLV*), diversity (*IGHD*), and joining (*IGHJ*, *IGKJ*, *IGLJ*) germline gene segments [2]. In addition, the imprecise junction of the germline gene segments and somatic hypermutations contribute to antibody diversity [3–5].

Species differences were primarily found in the number of germline *IGHV*/*IGKV*/*IGLV*, *IGHD*, and *IGHJ*/*IGKJ*/*IGLJ* segments. In livestock species with restricted combinatorial germline diversity such as chicken [4], pigs [6], sheep [7], and cattle [5, 8, 9], species-dependent mechanisms dominate the different diversification steps. For instance, the use of pseudogene sequence parts is a frequent post-recombinatorial strategy for the generation of the preimmune antibody repertoire in chicken, sheep, and rabbit [4, 10–13]. This phenomenon, called gene conversion, was also confirmed for IGLs in cattle [14] and is assumed to be operative in horses [15].

Gene conversions are difficult to detect especially within a large number of sequences e.g. like those obtained from high throughput sequencing. Gene conversion in immunoglobulins is characterized by clusters of nucleotide changes [14], sometimes only triplets [11], originating from upstream genes of the rearranged segment [4, 13]. High degree of flanking homology of the conversion region ensures the genetic exchange [13], whereby 3 to 5 nucleotides seem to be the minimal overlapping requirement [11].

Detection of gene conversion in bovine IGHV is complicated due to the incomplete IGH locus annotation. The main bovine IGH locus was assigned to the *Bos taurus* autosome (BTA) 21 but exons coding for variable, diversity, and joining segments were also found on BTA7, BTA8, and BTA20 [16–18]. *In silico* locus and mapping analyses identified 36 *IGHV*s of which nine are functional and belong phylogenetically to the bovine *IGHV* family 1 (boVH1). The second bovine *IGHV* family consists solely of non-functional *IGHV*s that have not been identified in expression analyses yet. Eleven *IGHV* segment pairs shared 100% sequence identity, whereas two of these pairs contain a functional segment and either an ORF or a putative functional segment, respectively [17]. The high proportion of pseudogene segments leads to the assumption of their use in gene conversion events. Two *IGHJ* loci possessing six *IGHJ* segments were detected on BTA11 by BAC clone and locus-specific PCR analysis and were found to rearrange at low frequency while those located on BTA21 rearrange at high frequency. Only two out of these six *IGHJ* were classified as functional whereas one is involved predominantly in the recombination process [19, 20]. Fifteen *IGHD* genes were detected and revealed a sub-cluster organization. *IGHD* are classified into four families and the *IGHD* exons revealed huge size differences [21, 22]. The organization of the actual bovine germline repertoire and its possible allelic variants is incomplete and needs to be investigated in more detail [17]. Since, even the organization of the extensively studied human immunoglobulin germline repertoire is questioned and requires ongoing analyses [23].

In all rearranged bovine immunoglobulin isotypes, exceptionally long complementarity determining region 3 of the heavy chain (CDR3H) possessing up to 67 aa were described [17]. Together with *IGHD2* and *IGHJ1*, the germline *IGHV10/34* segment was found to be the only variable segment rearranged in these exceptionally long CDR3Hs [17, 24], in a non-isotype dependent manner [17, 25]. Furthermore, those specialized CDR3H possessing several cysteine residues enabling the formation of intra-CDR3H disulfide bonds. Together with the C-terminal part of IGHV10/34, which forms an ascending β-strand, the CDR3H is consequently exposed like a knob like structure on top of the β-strand stalk whereby the descending β-strand is formed by the C-terminal IGHD portion. There are no similar structures described yet [26].

An additional bovine specific mechanism for antibody diversification is the insertion of conserved short nucleotide sequences into the *IGHV*-*IGHD* junction, which was found in intermediate and exceptionally long CDR3Hs [24].

Currently available programs like IMGT/Junction Analysis [27], IMGT/V-QUEST [28, 29] and IMTG/HIGHV-QUEST [30], VBASE2 [31], JoinSolver [32], iHMMun-align [33], and IgBLAST [34] allow the annotation of only the entire IGHV sequence to germline *IGHV* segments. Differentiated analysis of single parts is not directly possible. Most of the databases are focusing on mouse and human immunoglobulin genes (VBASE2, human, [31]). For cattle and other livestock or companion animals, separate databases have to be created (IgBLAST, [34]). Furthermore, the IMGT numbering system does not provide numbering for CDR3H larger than 31 aa. Placing of bovine intermediate as well as of exceptional long CDR3H in this numbering system is therefore not possible and consequently does not allow correct analysis of the rearrangement in those immunoglobulins. In addition, only IgBLAST allows the adjustment of parameters for *IGHD* identification.

Detailed genetic analysis of the pre- and post-immunization humoral immune response is important to describe the developing diversity and the effectiveness of vaccines and to detect possible individual and breed related differences including non-responders. As a conclusion those analyses help to develop fast recombinant antibodies for passive vaccination, therapy or diagnostic by genetically pre-selection of newly developed or abundant sequences.

As a first attempt to gain more insight into bovine breed specific differences, we compared the dominantly transcribed and the combinatorial diversity of germline *IGHV*, *IGHD*, and *IGHJ* segments as well as somatic hypermutations and putative gene conversions of IgG in the four cattle breeds Aubrac, German Simmental, German Black Pied and Holstein Friesian, by using a newly developed Bioinformatics framework. This new bioinformatics framework combines and extends several analysis tools and takes into account the unique specificities of bovine immunoglobulin sequences of exceptionally long CDR3Hs. In addition, it allows for the adaptation of alignment parameters for the single segments and enables the selective analysis of the different functional regions of the variable domain (namely framework regions and CDRs) to determine putative gene conversions. This new tool should facilitate a fast and detailed analysis of data sets generated by high throughput sequencing.

## Results

For sequence analysis, we developed a new bioinformatics framework using MUSCLE [35, 36] for the initial fast and accurate multiple nucleotide sequence alignment. Eventually, the sequence distances were calculated with ClustalW [37]. For nucleotide alignments of *IGHV* and *IGHJ*, default values of MUSCLE were used. To improve the biological significance of the assignment of germline and sample *IGHD*s, we tested three different procedures by changing parameters for gap opening/extension and including a scoring matrix for matches, transversions, and transitions. To determine the germline origin using the new bioinformatics framework, only the FRHs were aligned to avoid interference with the highly diversified CDRH [18]. To analyze possible gene conversion events, FR1-3Hs and CDR1-3Hs were extracted and aligned separately to the corresponding regions of the *IGHV* reference sequences to find the most similar germline segment.

For testing our bioinformatics tool, we established a sample sequence set for a detailed analysis of the transcribed bovine immunoglobulin repertoire. Blood samples were taken from 10 animals per cattle breed: Aubrac (A), German Simmental (GS), German Black Pied (GBP), and Holstein Friesian (HF).

In total, 160 IgG heavy chain sequences per breed (n = 640 sequences) were investigated as described above. The variable regions were identified and extracted at the 5’ end (N-terminal end) using the nucleotide motif GCCTCCACC coding for AlaSerThr marking the start of the first constant region of all bovine IgGs. Due to premature Stop-codons or incompletely amplified variable regions, 131 sequences were excluded from further analyses. Consequently, 509 sequences remained: 137 in A, 116 in GS, 111 in GBP, and 145 in HF. Sequences analyzed are published under accession numbers KT761498-KT762006.

### Transcriptional analyses, assignment of germline gene segments

#### Assigning the FR1H to FR3H of transcribed IGHV segments to their germline origin

**Fig 1:**
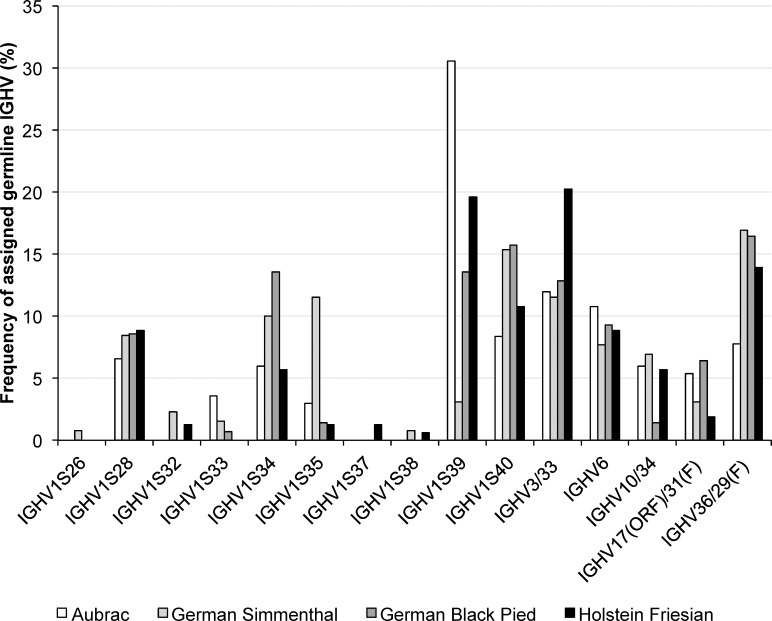
Transcription frequencies of *IGHV* in four cattle breeds. Transcribed IGHV are shown on the horizontal axis, their relative usage frequencies are indicated on the vertical axis. Each breed is marked by the following color code: Aubrac: white, German Simmental: light grey, German Black Pied: dark grey, Holstein Friesian: black

Ig heavy chain gene usage and identity to germline gene segments was determined by comparing the transcribed sequences with the germline Ig heavy chain genes described by Walther et al. [17] and Liljavirta et al. [22]. We found that 27 out of 62 germline *IGHV*s possess 100% nucleotide sequence identity (presented as *IGHVx/y*) up to the 3’ end of FR3H. Analysis of transcribed IGHV segments (here: comprising FR1-3H) revealed germline gene usage of *IGHV3/33*, *IGHV6*, *IGHV10/34*, *IGHV36/29(F)*, *IGHV17(ORF)/31(F)*, *IGHV1S26*, *IGHV1S28*, *IGHV1S32*, *IGHV1S33*, *IGHV1S34*, *IGHV1S35*, *IGHV1S37*, *IGHV1S38*, *IGHV1S39* as well as *IGHV1S40* (Fig 1).

We were not able to unambiguously assign ten of 116 analyzed GS sequences to one germline *IGHV*; this was also true for 14 out of 111 GBP sequences, 12 out of 145 HF sequences, and 19 out of 137 analyzed sequences of A. Samples showing equal divergence to at least two germline *IGHV* included combinations of *IGHV3/33*, *IGHV6*, *IGHV10/34*, *IGHV17(ORF)/31(F)*, *IGHV1S28*, and *IGHV1S32-40*. The most frequent multiple assignment affected *IGHV6* and *IGHV1S34*, followed by *IGHV6* and *IGHV1S35* in all A, GS, and HF. *IGHV6*, *IGHV1S34*, and *IGHV17(ORF)/31(F)* could not be differentiated in two sequences of GS, one sequence of GBP and two sequences of HF. Ambiguously assigned germline *IGHV*s and multiple assignments are listed in Table 1.

**Table 1:** *IGHV* assigned ambiguously and their frequency. Ambiguous sequences can be assigned to more than one germline *IGHV* with the same distance.

Breed	A	GBP	GS	HF
Total No. of animals	10	10	10	10
No. of animals with ambiguous sequences	9	5	4	6
Total No. of sequences analyzed	137	111	116	145
No. of ambiguous sequences	19	14	10	12
Total No. of putative *IGHV* recombinations	167	140	130	158
**Ambiguous *IGHV***				
*IGHV3/33/IGHV1S33*	0	0	1	0
*IGHV3/33/IGHV1S39*	0	0	0	2
*IGHV6/IGHV17(ORF)/31(F)*	1	0	0	0
*IGHV6/IGHV1S34*	3	3	3	5
*IGHV6/IGHV1S35*	3	0	1	1
*IGHV6/IGHV36/29(F)*	0	1	1	1
*IGHV6/IGHV1S39*	1	0	1	0
*IGHV1S28/IGHV1S32*	0	0	0	1
*IGHV1S33/IGHV1S39*	1	1	0	0
*IGHV1S33/IGHV1S40*	1	0	0	0
*IGHV1S37/IGHV1S38*	0	0	0	1
*IGHV1S39/IGHV17(ORF)/31(F)*	0	1	0	0
*IGHV1S39/IGHV1S40*	1	0	0	0
*IGHV3/33/IGHV10/34/IGHV1S39*	2	0	0	0
*IGHV6/IGHV17(ORF)/31(F)/IGHV1S34*	0	2	2	1
*IGHV6/IGHV17(ORF)/31(F)/IGHV1S40*	1	0	0	0
*IGHV6/IGHV1S32/IGHV1S34*	1	0	1	0
*IGHV6/IGHV1S33/IGHV1S35*	1	0	0	0
*IGHV3/33/IGHV6/IGHV17(ORF)/31(F)/IGHV1S34*	0	2	0	0
*IGHV6/IGHV10/34/IGHV1S34/IGHV1S39*	1	0	0	0
*IGHV6/IGHV17(ORF)/31(F)/IGHV1S28/IGHV1S39*	0	1	0	0
*IGHV6/IGHV17(ORF)/31(F)/IGHV1S34/IGHV1S39*	1	1	0	0
*IGHV6/IGHV36/29(F)/IGHV1S34/IGHV1S39*	0	1	0	0
*IGHV3/33/IGHV6/IGHV17(ORF)/31(F)/IGHV1S34/IGHV1S39*	0	1	0	0
*IGHV6/IGHV17(ORF)/31(F)/IGHV1S33/IGHV1S34/IGHV1S35*	1	0	0	0

Including the multiple assignments as described above, a total number of 595 possible transcribed germline *IGHV* were observed. Overall, the most frequent variable gene segment was *IGHV1S39*; this was identified in 17.65% of all sequences. This *IGHV* was used in 51 A sequences (n = 167, 30.54%), in 19 sequences of GBP (n = 140, 13.57%), in four sequences of GS (n = 130, 3.08%), and 31 sequences of HF (n = 158, 19.62%) (Fig 1, Table 2). *IGHV3/33* was represented by 14.29% of all sequences. The number of transcribed *IGHV3/33* varied from 15 in GS (11.54%) to 32 in HF (20.25%) (Fig 1, Table 2). In similar proportions of 13.45% and 12.27% germline *IGHV36/29(F)* and *IGHV1S40* were used. *IGHV1S28*, *IGHV1S34*, and *IGHV6* were transcribed in proportions of 8.07%, 8.57%, and 9.24%. The other transcribed IGHVs were identified in minor proportions of 0.17% to 5.04%, respectively. These rarely used *IGHV*s were identified once or twice in GS and HF but up to ten times in A (Fig 1). Very high significant differences were calculated for *IGHV* usage within the breeds and between the breeds (P<0.0001).

**Table 2:** Percentage of *IGHV* assigned to sample sequences of four cattle breeds.

*IGHV*	A(%; n = 167)	GS(%; n = 130)	GBP(%; n = 158)	HF(%; n = 140)
*IGHV1S26*	0.00	0.77	0.00	0.00
*IGHV1S28*	6.59	8.46	8.57	8.86
*IGHV1S32*	0.00	2.31	0.00	1.27
*IGHV1S33*	3.59	1.54	0.71	0.00
*IGHV1S34*	5.99	10.00	13.57	5.70
*IGHV1S35*	2.99	11.54	1.43	1.27
*IGHV1S37*	0.00	0.00	0.00	1.27
*IGHV1S38*	0.00	0.77	0.00	0.63
*IGHV1S39*	30.54	3.08	13.57	19.62
*IGHV1S40*	8.38	15.38	15.71	10.76
*IGHV3/33*	11.98	11.54	12.86	20.25
*IGHV6*	10.78	7.69	9.29	8.86
*IGHV10/34*	5.99	6.92	1.43	5.70
*IGHV17(ORF)/31(F)*	5.39	3.08	6.43	1.90
*IGHV36/29(F)*	7.78	16.92	16.43	13.92

#### Separate analyses of FRH 1–3 and CDRH 1–2 to detect gene conversion events

In livestock such as chicken, rabbit, and cattle the use of pseudogene segments is known to contribute to immunoglobulin diversity [4, 10, 11, 13, 14, 38, 39]. Gene conversion in immunoglobulins is characterized by clusters of nucleotide changes [14], sometimes only triplets [11], originating from upstream genes of the rearranged segment [4, 13]. A high degree of flanking homology of the conversion region ensures the genetic exchange [13], whereby three to five nucleotides have been shown to be the minimal overlapping requirement [11].

Consequently, FR1-3H and CDR1-2H were analyzed separately to identify mutations within the FR1-3H and CDR1-2H that would indicate possible gene conversion events. The current genomic annotation of germline immunoglobulin segments in cattle makes a correct prediction of 5’ donor segments difficult. Nonetheless, larger contigs have been identified [17] and enabled us to show gene conversion events in bovine immunoglobulin heavy chains variable regions.

For instance, the calculated putative originating germline genes for nucleotide changes identified solely in the CDR2H region of KT761864 were *IGHV4Ψ/32Ψ*, *IGHV9Ψ/35Ψ*, and *IGHV18Ψ/30Ψ* (divergence 0.190). In the calculation covering the whole V-region and using only FR1-3H, *IGHV6* was identified as the originating gene for KT761864 (divergence 0.075). All genes but *IGHV18Ψ/30Ψ* are located on BTA21, whereas *IGHV4Ψ/32Ψ* is located upstream of *IGHV6* and is therefore most likely used for the gene conversion. There are two triplets in KT761864 and *IGHV4Ψ/32Ψ* that are different from *IGHV6* due to a transversion mutation in the first changed triplet (from AAT in *IGHV6* to TAT in KT761864 and *IGHV4Ψ/32Ψ*) and a transition mutation in the second changed triplet (from GAT in *IGHV6* to AAT in KT761864 and *IGHV4Ψ/32Ψ*; Table 3).

**Table 3:** Possible gene conversion in KT761864.

Sample/Gene	Sequence in CDR2H	Location	Divergence
KT761864	ATT AAT TAT AAT … GGA GAC ACC	BTA21	
*IGHV4Ψ/32Ψ*	ATA TAT TAT AAT … GGT GAC ACT	BTA21	0.190
*IGHV9Ψ/35Ψ*	ATA TAT TAT AAT … GGT GAC ACT	BTA21	0.190
*IGHV18Ψ/30Ψ*	ATA TAT TAT AAT … GGT GAC ACT	BTA7	0.190
*IGHV6*^*1*^	ATA GAT AAT GAT … GGA GAC ACA	BTA21	0.075

^1^ Divergence in calculations covering the whole V-region and using only FR1-3H. This gene was not observed in the analysis of the separated CDR2H.

Due to high sequence similarities between functional and pseudogenes, we initially assumed the preferable transcription of functional germline gene segments. If none of the functional germline segments was assigned as origin, we concentrated on pseudogenes that were calculated as unique source of the sample sequences considering the particular regions.

**Fig 2:**
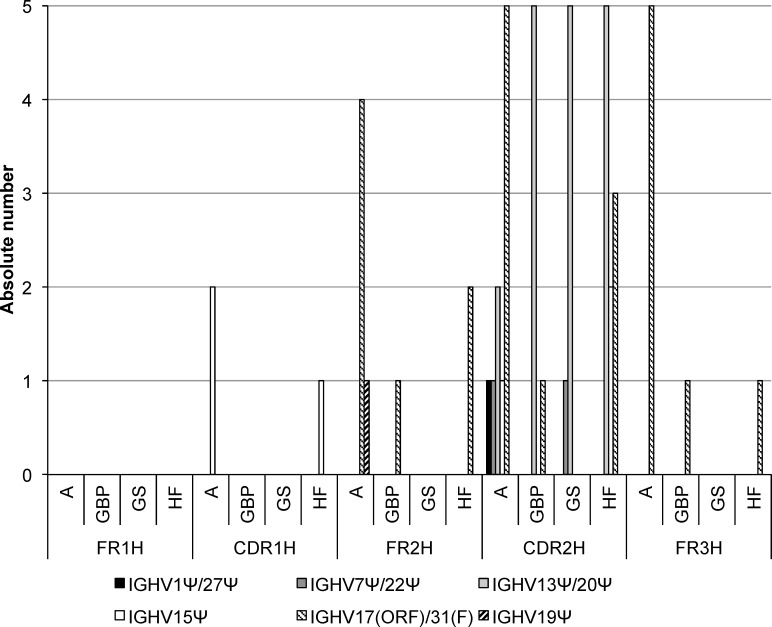
Possible gene conversion in FRH and CDRH. The absolute number of possible gene conversion events is shown for pseudogenes assigned unambiguously to CDR1H, FR2H, and CDR2H in the four cattle breeds Aubrac (A), German Black Pied (GBP), German Simmental (GS), and Holstein Friesian (HF).

In all breeds, *IGHV13Ψ/20Ψ* and *IGHV17(ORF)/31(F)* were used most often in CDR2H (3.34% and 1.77%) as well as in FR2H and FR3H (*IGHV17(ORF)/31(F)* both 1.38%) and CDR2H (*IGHV13Ψ/20Ψ* 1.77%), respectively. *IGHV15Ψ* was found in 0.59% of CDR2H in sequences of A and HF. *IGHV11Ψ*, *IGHV12Ψ*, and *IGHV21Ψ* always showed the same divergence from GBP and HF sequences but were not solely identified as the potential origin of the sample sequence. Similarly, *IGHV4Ψ/32Ψ*, *IGHV9Ψ/35Ψ*, and *IGHV18Ψ/30Ψ* were identified in triplet as possible originating gene segments but only together with functional germline genes. In one sequence of GBP and two sequences of GS both in CDR1H and CDR2H pseudogenes were calculated as possible parental *IGHV*s. In one A sequence only a pseudogene showed lowest divergence from the sample in FR2H and CDR2H (Fig 2).

Our separate analyses of FRHs and of CDRHs revealed a less unambiguous assignment to the germline gene segments than in the previous analysis of the entire *IGHV*. Nevertheless, 97.65% of identified germline sequences were *IGHV3/33*, *IGHV6*, *IGHV10/34*, *IGHV2/26*, *IGHV29(F)/36*, *IGHV16(ORF)/25*, *IGHV17(ORF)/31(F)*, and *IGHV19Ψ*, as well as *IGHV1S19*, *IGHV1S23-28*, *IGHV1S30*, *IGHV1S32-40* [22]. Although all of these are the same gene segments as determined in the analyses of the complete IGHV, our calculations indicate nucleotide sequence exchanges between *IGHV*s within FRHs and CDRHs. Our bioinformatics tool identified additional pseudogenes, *IGHV1Ψ/27Ψ*, *IGHV4Ψ/32Ψ*, *IGHV7Ψ/22Ψ*, *IGHV9Ψ/35Ψ*, *IGHV11Ψ*, *IGHV12Ψ*, *IGHV13Ψ/20Ψ*, *IGHV15Ψ*, *IGHV18Ψ/30Ψ*, *IGHV21Ψ*, predominantly by CDR1H and CDR2H analysis. *IGHV13Ψ/20Ψ*, *IGHV15Ψ*, and *IGHV19Ψ* showed little divergence in FR1H and FR2H from sample sequences, but different functional *IGHVs* were also calculated as possible originating germline sequences (Table 4).

**Table 4:** Possible gene conversions in FRH and CDRH.

		A		GBP		GS		HF		Sum		Percent
		a^1^	u^2^	a^1^	u^2^	a^1^	u^2^	a^1^	u^2^	a^1^	u^2^	u^2^
*IGHV1Ψ/27Ψ*	CDR1H	1	0	0	0	0	0	1	0	2	0	0.00
	CDR2H	1	1	1	0	1	0	2	0	5	1	0.20
*IGHV4Ψ/32Ψ*	CDR1H	1	0	0	0	0	0	1	0	2	0	0.00
	CDR2H	4	0	1	0	2	0	2	0	9	0	0.00
*IGHV7Ψ/22Ψ*	CDR2H	2	1	1	0	1	1	4	0	8	2	0.39
*IGHV9Ψ/35Ψ*	CDR1H	1	0	0	0	0	0	1	0	2	0	0.00
	CDR2H	4	0	1	0	2	0	2	0	9	0	0.00
*IGHV11Ψ*	CDR2H	0	0	0	0	0	0	2	0	2	0	0.00
*IGHV12Ψ*	CDR2H	0	0	0	0	0	0	2	0	2	0	0.00
*IGHV13Ψ/20Ψ*	CDR1H	18	0	11	0	9	0	19	0	57	0	0.00
	FR2H	1	0	0	0	0	0	0	0	1	0	0.00
	CDR2H	16	2	20	5	17	5	22	5	75	17	3.34
*IGHV17(ORF)/31(F)*	FR1H	80	0	58	0	63	0	75	0	276	0	0.00
	CDR1H	20	0	10	0	2	0	10	0	42	0	0.00
	FR2H	8	4	4	1	4	0	6	2	22	7	1.38
	CDR2H	12	5	7	1	3	0	14	3	36	9	1.77
	FR3H	9	5	10	1	9	0	8	1	36	7	1.38
*IGHV15Ψ*	CDR1H	7	2	3	0	3	0	4	1	17	3	0.59
	FR2H	0	0	0	0	0	0	0	0	0	0	0.00
	CDR2H	3	1	9	0	1	0	9	2	22	3	0.59
*IGHV18Ψ/30Ψ*	CDR1H	1	0	0	0	0	0	1	0	2	0	0.00
	CDR2H	4	0	1	0	2	0	2	0	9	0	0.00
*IGHV19Ψ*	FR1H	80	0	58	0	63	0	75	0	276	0	0.00
	CDR1H	25	0	13	0	27	0	20	0	85	0	0.00
	FR2H	6	1	1	0	0	0	4	0	11	1	0.20
	CDR2H	9	0	15	0	9	0	25	0	58	0	0.00
*IGHV21Ψ*	CDR2H	0	0	0	0	0	0	2	0	2	0	0.00

^1^ ambiguously assigned

^2^ unambiguously assigned

#### CDR3H length distribution

**Fig 3:**
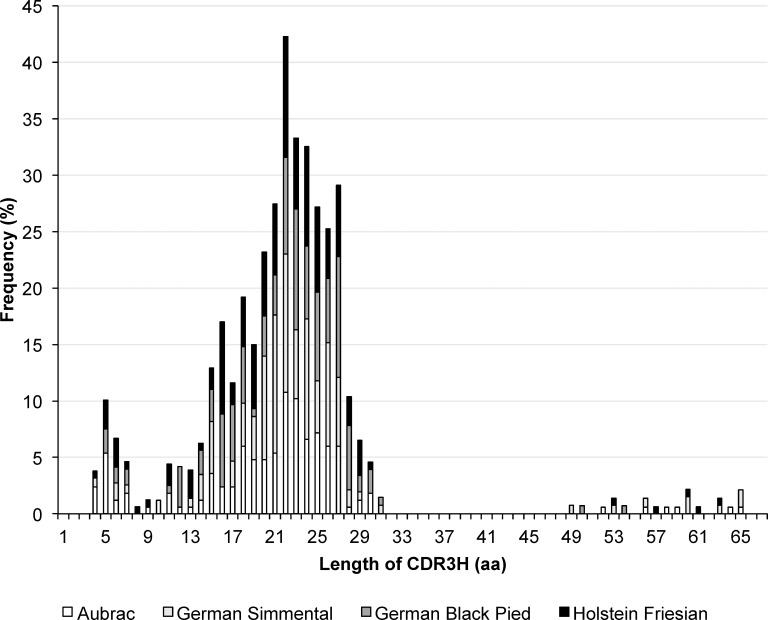
Length distribution of CDR3H in the four cattle breeds. The number of amino acid residues making up the CDR3H lengths identified are shown on the horizontal axis, their relative usage frequencies are indicated on the vertical axis. Each breed is marked by the following color code: Aubrac: white, German Simmental: light grey, German Black Pied: dark grey, Holstein Friesian: black

In all four cattle breeds the program identified very short CDR3Hs (less or equal 10 aa, group 1), CDR3Hs of intermediate length (11–47 aa, group 2) as well as exceptionally long CDR3Hs (at least 48 aa, group 3) (Fig 3, Table 5). Very high significant differences were calculated for the number of sequences within the 3 groups of lengths (P<0.0001) when they were compared between all breeds but also within the breeds (GS, GBP, A, HF: P<0.0001). Fourty-four (7.37%) sequences possessed a CDR3H length with ten or less amino acid (aa) residues. The highest amount of sequences within this group were identified in breed A (12.57%), followed by HF (7.55%), GBP (5.00%) and GS (3.05%). In the breeds A, GS, and HF one and four sequences possessed only four amino acid residues within the CDR3H. Five amino acid residues were the shortest CDR3H in sequences of GBP.

**Table 5:** Length distribution of the CDR3Hs.

Number of amino acids within CDR3H	All breeds (%; n = 597)	GS (%; n = 131)	GBP (%; n = 140)	HF (%; n = 159)	A (%; n = 167)
4	1.01	0.76	0.00	0.63	2.40
5	2.68	0.00	2.14	2.52	5.39
6	1.68	1.53	1.43	2.52	1.20
7	1.17	0.76	1.43	0.63	1.80
8	0.17	0.00	0.00	0.63	0.00
9	0.34	0.00	0.00	0.63	0.60
10	0.34	0.00	0.00	0.00	1.20
11	1.17	0.00	0.71	1.89	1.80
12	1.01	0.00	3.57	0.00	0.60
13	1.01	0.76	0.00	2.52	0.60
14	1.51	2.29	2.14	0.63	1.20
15	3.18	4.58	2.86	1.89	3.59
16	4.36	0.00	6.43	8.18	2.40
17	2.85	2.29	5.00	1.89	2.40
18	4.86	3.82	5.00	4.40	5.99
19	3.85	3.82	0.71	5.66	4.79
20	5.70	9.16	3.57	5.66	4.79
21	6.70	12.21	3.57	6.29	5.39
22	10.55	12.21	8.57	10.69	10.78
23	8.38	6.11	10.71	6.29	10.18
24	8.04	10.69	6.43	8.81	6.59
25	6.87	4.58	7.86	7.55	7.19
26	6.20	9.16	5.71	4.40	5.99
27	7.20	6.11	10.71	6.29	5.99
28	2.51	1.53	5.71	2.52	0.60
29	1.68	0.76	1.43	3.14	1.20
30	1.17	0.00	2.14	0.63	1.80
31	0.34	0.76	0.71	0.00	0.00
49	0.17	0.76	0.00	0.00	0.00
50	0.17	0.00	0.71	0.00	0.00
52	0.17	0.00	0.00	0.00	0.60
53	0.34	0.76	0.00	0.63	0.00
54	0.17	0.00	0.71	0.00	0.00
56	0.34	0.76	0.00	0.00	0.60
57	0.17	0.00	0.00	0.63	0.00
58	0.17	0.00	0.00	0.00	0.60
59	0.17	0.00	0.00	0.00	0.60
60	0.50	1.53	0.00	0.63	0.00
61	0.17	0.00	0.00	0.63	0.00
63	0.34	0.76	0.00	0.63	0.00
64	0.17	0.00	0.00	0.00	0.60
65	0.50	1.53	0.00	0.00	0.60

CDR3H of group 2 were identified in 532 of all sequences analyzed (89.11%). 93.57% of GBP sequences were found to use 11 up to 47 aa. In HF, 89.31% of the sequences were attributed to this group as well as 83.83% of A and 90.84% of GS sequences. The most frequent CDR3H length was 22 aa, which was found in 10.78% of A sequences, in 8.57% of GBP, in 12.21% of GS, and in 10.69% HF sequences. Simultaneously, this length was identified preferably in CDR3H of A and HF. Nevertheless, in GS CDR3H with a length of 21 aa was identified as often as a length of 22 aa (12.21%). In GBP, CDR3Hs with a length of 23 and 27 aa dominated (10.71%).

CDR3Hs of group 3 were identified in 21 sequences of all four breeds (9.22%). The breed GS showed the highest number of these sequences (6.11%) followed by A (3.59%). German Black Pied and HF sequences possessed smaller proportions of the exceptionally long CDR3H with 3.14% and 1.43%, respectively. Whereas in GS and HF sequences with 65 aa were the longest CDR3Hs (1.53%, 0.6%), 63 and 54 aa were counted in the longest CDR3Hs of A (0.63%) and GBP (0.71%), respectively.

#### Assigning IGHD to their germline origin using 3 different procedures

We tested three different procedures to assign germline and sample *IGHD*s in order to improve the biological significance. At first, we applied the default values of MUSCLE, in procedure 2 we changed the penalties for gap opening (= -4) and gap extention (= -0.3), and in procedure 3 we additionally incorporated a modified scoring matrix (match +2) to evaluate transversion and transition mutations.

**Fig 4:**
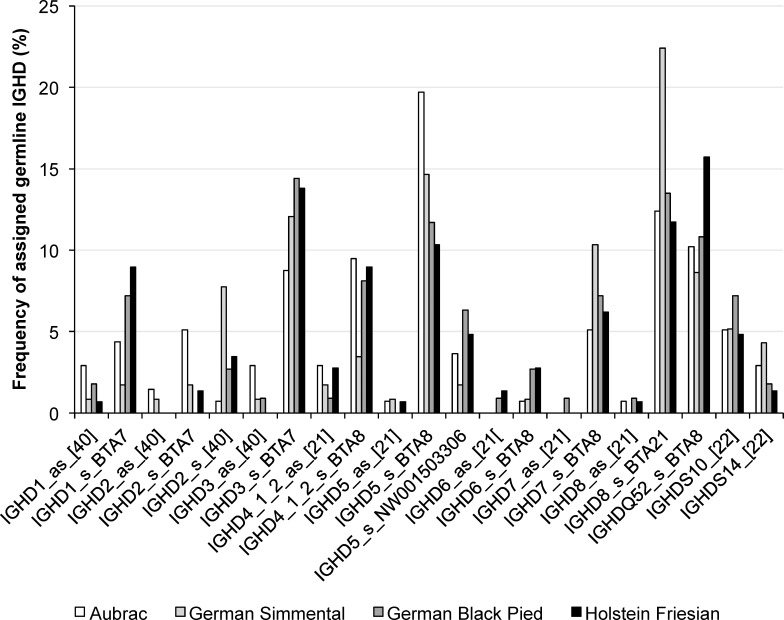
Transcription frequencies of *IGHD* in four cattle breeds using procedure 3. Transcribed IGHD are shown on the horizontal axis, their relative usage frequencies are indicated on the vertical axis. Calculation occurred after changing the default values for gap opening (-4) and gap extention (-0.3) and a modified scoring matrix (match +2) of MUSCLE. Each breed is marked by the following color code: Aubrac: white, German Simmental: light grey, German Black Pied: dark grey, Holstein Friesian: black

In all three procedures, the assignment of germline and transcribed *IGHD* revealed clear results for the sequences analyzed. Nevertheless, using procedure 3 we obtained results matching short and long sample IGHD sequences best to germline short CDR3H and exceptionally long CDR3H, respectively, whereas procedure 1 and 2 assigned a major number of group 3 CDR3H to germline *IGHD* of moderate length. Twenty-one different germline *IGHD* were transcribed, whereby *IGHD8* located on BTA21, *IGHD3* located on BTA7, *IGHD5* as well as the very short *IGHDQ52* located on BTA8; these were preferred in all breeds (Fig 4, Table 6). While *IGHDQ52*, *IGHDS10* and *14* [22] were solely transcribed in sense orientation, germline *IGHD1* to *IGHD8* gene segments were transcribed in antisense direction in 38 sequences distributed over all four breeds investigated. *IGHD4* (antisense (as), [21]) was identified the most often, followed by *IGHD1* (as, [40]), *IGHD3* (as, [40]), *IGHD2* (as, [40]), *IGHD5* (as, [21]), *IGHD6* (as, [21]), *IGHD8* (as, [21]) and *IGHD7* (as, [21]). Very high significant differences were calculated for the *IGHD* usage within the cattle breeds (P<0.0001) but not between the breeds (P = 0.06).

**Table 6:** Percentage of *IGHD* assigned to sample sequences of four cattle breeds.

IGHD	A (%; n = 137)	GS (%; n = 116)	GBP (%; n = 111)	HF (%; n = 145)
*IGHD1*_as^1^_[39]	2.92	0.86	1.80	0.69
*IGHD1*_s^2^_BTA7	4.38	1.72	7.21	8.97
*IGHD2*_as_[39]	1.46	0.86	0.00	0.00
*IGHD2*_s_BTA7	5.11	1.72	0.00	1.38
*IGHD2*_s_[39]	0.73	7.76	2.70	3.45
*IGHD3*_as_[39]	2.92	0.86	0.90	0.00
*IGHD3*_s_BTA7	8.76	12.07	14.41	13.79
*IGHD4*_as_[21]	2.92	1.72	0.90	2.76
*IGHD4*_s_BTA8	9.49	3.45	8.11	8.97
*IGHD5*_as_[21]	0.73	0.86	0.00	0.69
*IGHD5*_s_BTA8	19.71	14.66	11.71	10.34
*IGHD5*_s_NW001503306	3.65	1.72	6.31	4.83
*IGHD6*_as_[21]	0.00	0.00	0.90	1.38
*IGHD6*_s_BTA8	0.73	0.86	2.70	2.76
*IGHD7*_as_[21]	0.00	0.00	0.90	0.00
*IGHD7*_s_BTA8	5.11	10.34	7.21	6.21
*IGHD8*_as_[21]	0.73	0.00	0.90	0.69
*IGHD8*_s_BTA21	12.41	22.41	13.51	11.72
*IGHDQ52*_s_BTA8	10.22	8.62	10.81	15.71
*IGHDS10*_[22]	5.11	5.17	7.21	4.83
*IGHDS14*_[22]	2.92	4.31	1.80	1.38

^1^ antisense

^2^ sense

In comparison, 20 different germline *IGHD* were transcribed using default conditions of MUSCLE for alignments (procedure 1), whereby *IGHD8* being located on BTA21 was the preferred *IGHD* in all breeds. The very short *IGHDQ52* located on BTA8 was transcribed in high frequencies in the breeds A, GBP, and HF (S1 Fig, S1 Table). While *IGHDQ52*, *IGHDS10* and *14*, and *IGHD6* were solely transcribed in sense orientation, germline *IGHD1* to *IGHD8* gene segments were transcribed in antisense direction in 23 sequences that were distributed over all four breeds. *IGHD4* (as, [21]) was identified the most often. Very high significant differences were calculated for the *IGHD* usage within the cattle breeds (P<0.0001) but not between the breeds (P = 0.1630).

After changing penalties for gap opening and gap extension (procedure 2), 17 different germline *IGHD* were transcribed, whereby *IGHD8* located on BTA21, was the most observed *IGHD* in all breeds (S2 Fig, S2 Table). Germline *IGHD1*, *IGHD4*, *IGHD6*, and *IGHD8* gene segments were transcribed in as direction in 24 sequences that were distributed over all four breeds. Again, *IGHD4* (as, [21]) was identified the most often. Very high significant differences were calculated for the *IGHD* usage within the cattle breeds (P<0.0001) but not between the breeds (P = 0.6654).

#### Assigning the FR4H and IGHJ to their germline origin

**Fig 5:**
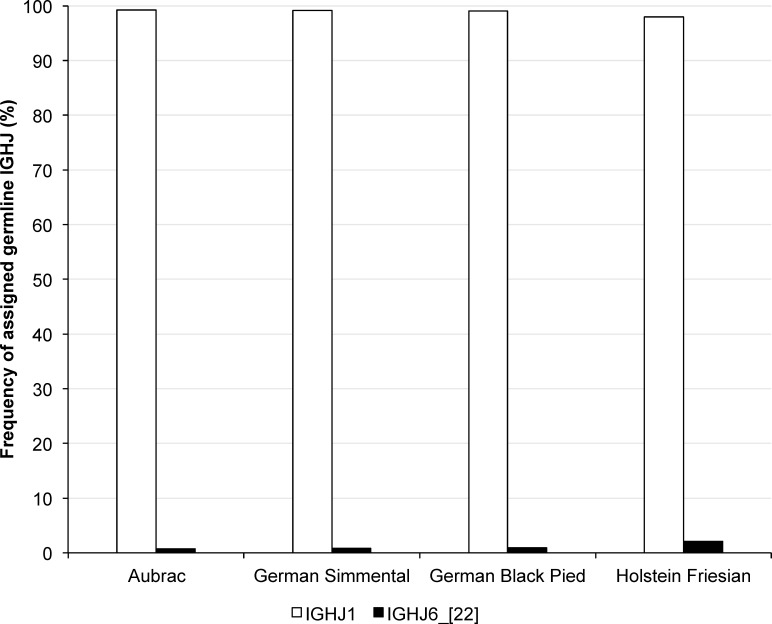
Transcription frequencies of *IGHJ* in four cattle breeds. Transcribed IGHJ are shown on the horizontal axis, their relative usage frequencies are indicated on the vertical axis. Each breed is marked by the following color code: Aubrac: white, German Simmental: light grey, Holstein Friesian: black, German Black Pied: dark grey

Located on BTA21, *IGHJ1* and *IGHJ6* [22] were identified as origin to the transcribed gene segments, which defines the FR4H within the samples analyzed. *IGHJ1* was transcribed preferably in the sequences investigated in all breeds (98.83%). *IGHJ6* [22] (1.17%) was detected in only one sequence of each A, GS, and GBP (0.73%, 0.85%, 0.90%), as well as in three sequences of HF (2.05%) animals. One sequence of GS and HF, respectively, showed *IGHJ1* and *IGHJ6* as a possible originating germline segment (Fig 5, Table 7). Statistical analysis revealed very high significant differences (P<0.0001) for the usage of *IGHJ1* and *IGHJ6* [22] within all cattle breeds. No significant differences were calculated between the four breeds.

**Table 7:** Percentage of *IGHJ* assigned to sample sequences of four cattle breeds.

*IGHJ*	A (%; n = 137)	GS (%; n = 117)	GBP (%; n = 111)	HF (%; n = 146)
*IGHJ1*	99.27	99.15	99.10	97.95
*IGHJ6* [22]	0.73	0.85	0.90	2.05

#### Recombination of IGHV, IGHD, and IGHJ in different cattle breeds using procedure 3

Recombined *IGHV*, *IGHD*, and *IGHJ* were identified for each sequence and all possible frequencies were analyzed statistically within and between the breeds examined. In total, 597 recombinations were analyzed including double assigned germline origins for *IGHV* and *IGHJ*.

**Fig 6:**
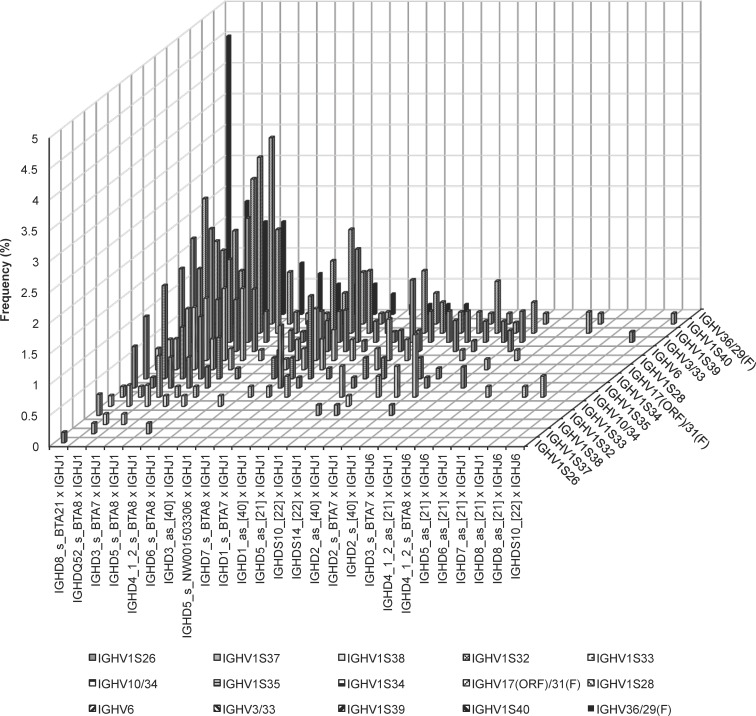
Recombinations of *IGHV*, *IGHD*, and *IGHJ* over all four cattle breeds. In the sequences of all four cattle breeds analyzed 162 different combinations of *IGHV*, *IGHD*, and *IGHJ* were identified. Relative frequencies (%) of the combinations of the 21 transcribed *IGHD* and the 2 transcribed *IGHJ* are shown depending on the rearranged *IGHV* (n = 15).

Applying procedure 3 for the *IGHD* assignment, 162 different combinations of *IGHV*, *IGHD*, and *IGHJ* were revealed (Fig 6, S3 Table). Most combinations occurred in less than ten sequences. Calculations revealed very high significant differences in usage frequencies of the rearranged gene segments between the breeds investigated (P<0.0001). Eleven rearrangements were observed in ten to 27 sequences. They were observed in sequences of all four breeds. These major rearrangements were: IGHV36/29(F)-IGHD8 (sense (s), BTA21)-IGHJ1 (AY158087) (4.52%), IGHV1S40-IGHD5 (s, BTA8)-IGHJ1 (AY158087) (3.02%), IGHV1S39-IGHD5 (s, BTA8)-IGHJ1 (AY158087) (2.85%), IGHV1S40-IGHD3 (s, BTA7)-IGHJ1 (AY158087) (2.35%), IGHV1S39-IGHD8 (s, BTA21)-IGHJ1 (AY158087) (2.18%), IGHV3/33-IGHD5 (s, BTA8)-IGHJ1 (AY158087) (2.01%), IGHV3/33-IGHDQ52 (s, BTA8)-IGHJ1 (AY158087) (1.84%), IGHV36/29(F)-IGHDQ52 (s, BTA8)-IGHJ1 (AY158087) (1.84%), IGHV1S39-IGHD4 (s, BTA8)-IGHJ1 (AY158087) (1.68%), IGHV1S39-IGHD7 (s, BTA8)-IGHJ1 (AY158087) (1.68%), IGHV3/33-IGHD8 (s, BTA21)-IGHJ1 (AY158087) (1.68%). Beside these preferred combinations, 52 minor recombinations were identified solely in one sequence distributed with 19 sequences in A, 14 in GS, six in GBP, and 13 in HF. Six variations of rearranged *IGHJ6* were also identified one and two-times. These appear distributed over all four breeds: IGHV3/33-IGHD3 (s, BTA7)-IGHJ6 [22], IGHV1S28-IGHD4 (s, BTA8)-IGHJ6 [22], IGHV1S39-IGHD3 (s, BTA7)-IGHJ6 [22], IGHV1S40-IGHDS10-IGHJ6 [22], IGHV3/33-IGHD8 (as, [21])-IGHJ6 [22], as well as IGHV10/34-IGHD5 (as, [21])-IGHJ6 [22].

Fifteen out of the 162 IGHV-IGHD-IGHJ combinations were identified in all four cattle breeds investigated. In animals of A, 91 different rearrangements were found, whereas in GBP 74 different recombinations were observed. German Simmental revealed 72 combinations and HF showed 85 variations. Within the cattle breed A (167 rearrangements), the combinations IGHV1S39-IGHD5 (s, BTA8)-IGHJ1 (AY158087), IGHV1S39-IGHD8 (s, BTA21)-IGHJ1 (AY158087), and IGHV1S39-IGHD4 (s, BTA8)-IGHJ1 (AY158087) were most frequently used in 7.78%, 4.79%, and 3.59%, respectively. Chi square calculations revealed high significant differences in usage frequencies of the recombinations in the breed A (P = 0.0004). In animals of HF (159 rearranged sample sequences), the dominant rearrangements were IGHV3/33-IGHD8 (s, BTA21)-IGHJ1 (AY158087) (4.40%), IGHV3/33-IGHDQ52 (s, BTA8)-IGHJ1 (AY158087) (3.77%), IGHV36/29(F)-IGHDQ3 (s, BTA7)-IGHJ1 (AY158087) (3.77%), IGHV3/33-IGHD5 (s, BTA8)-IGHJ1 (AY158087) (3.14%), and IGHV3/33-IGHD1 (s, BTA7)-IGHJ1 (AY158087) (3.14%). The Chi square test did not show significant usage differences (P = 0.6689) of the different rearranged gene segments in this breed. Very high significant differences were calculated for the recombined IGHV-IGHD-IGHJ in the breeds GS (P<0.0001), while in GBP no significant differences were found (P = 0.8870). In GS animals rearranged IGHV36/29(F)-IGHD8 (s, BTA21)-IGHJ1 (AY158087) (10.69%), IGHV1S40-IGHD3 (s, BTA7)-IGHJ1 (AY158087) (4.58%), IGHV1S40-IGHD5 (s, BTA8)-IGHJ1 (AY158087) (4.58%), IGHV1S34-IGHD8 (s, BTA21)-IGHJ1 (AY158087) (3.82%), and IGHV1S35-IGHD7 (s, BTA8)-IGHJ1 (AY158087) (3.82%) were observed in at least five sequences (131 rearranged samples). Four rearrangements were preferred in GBP (140 rearranged sequences): IGHV36/29(F)-IGHD8 (s, BTA21)-IGHJ1 (AY158087) (5.00%), IGHV1S40-IGHD5 (s, BTA8)-IGHJ1 (AY158087) (3.57%), and IGHV36/29(F)-IGHDQ52 (s, BTA8)-IGHJ1 (AY158087) (3.57%), and IGHV1S34-IGHD1 (s, BTA7)-IGHJ1 (AY158087) (3.57%).

#### Recombination of IGHV, IGHD, and IGHJ in different cattle breeds using procedure 1

With the alignment conditions applied in procedure 1 and 2, different predominantly used recombinations were observed as expected due to the different identified *IGHD*s. Using the default values of MUSCLE (procedure 1), 147 different combinations of *IGHV*, *IGHD*, and *IGHJ* were found (S3 Fig, S4 Table). Most combinations occurred in less than ten sequences. We found very high significant differences in the usage frequencies of the rearranged gene segments between the breeds investigated (P<0.0001). We also observed rearrangements observed in a quantity of ten to 21 sequences that also occurred in sequences spanning of all four breeds. The major rearrangement was IGHV36/29(F)-IGHD8 (s, BTA21)-IGHJ1 (AY158087) (3.52%). Beside this preferred combination, 49 were identified solely in one sequence, whereby 12 were identified in A, 15 in GS, nine in GBP, and 13 in HF. We also identified seven variations rearranging *IGHJ6* over all animals.

Twenty-one out of the 147 IGHV-IGHD-IGHJ combinations were identified in all four cattle breeds investigated. In animals of A, 81 different rearrangements were found, whereas in GBP 74 different recombinations were observed. German Simmental had 74 combinations and HF had 80 variations. Within the cattle breed A (167 rearranged sample sequences), the combination IGHV1S39-IGHD3 (s. BTA7)-IGHJ1 (AY158087) was the most frequently used in 5.99%. Chi square calculations revealed significant differences in usage frequencies of the recombinations in the breed A (P = 0.0108). In animals of HF (159 rearranged sample sequences), the dominant rearrangement was IGHV1S40-IGHD5 (s, BTA8)-IGHJ1 (AY158087) (4.40%). The Chi square test did not show significant usage differences (P = 0.0546) for the different rearranged gene segments in this breed. High significant differences were seen for the recombined IGHV-IGHD-IGHJ in the breeds GS (P = 0.0002), while in GBP no significant difference was found (P = 0.9585). In GS animals rearranged IGHV36/29(F)-IGHD8 (s, BTA21)-IGHJ1 (AY158087) (9.16%) was observed in at least six sequences (131 rearranged samples). Four rearrangements were preferred in GBP (140 rearranged sequences): IGHV36/29(F)-IGHD8 (s, BTA21)-IGHJ1 (AY158087) (3.57%), IGHV1S40-IGHD8 (s, BTA8)-IGHJ1 (AY158087) (3.57%), IGHV1S34-IGHD5 (s, BTA8)-IGHJ1 (AY158087) (3.57%), and IGHV36/29(F)-IGHD5 (s, NW_001503306)-IGHJ1 (AY158087) (3.57%).

#### Recombination of IGHV, IGHD, and IGHJ in different cattle breeds using procedure 2

When applying procedure 2 with changed values for gap opening and gap extension, we identified 119 different combinations of *IGHV*, *IGHD*, and *IGHJ* (S4 Fig, S5 Table) were identified. As seen for the other procedures, most combinations occurred in less than ten sequences. Calculations revealed very high significant differences in usage frequencies of the rearranged gene segments between the breeds investigated (P<0.0001). Fifteen different rearrangements were observed in a quantity of ten to 42 sequences and were observed across all four breeds. The major rearrangement was IGHV1S39-IGHDQ52 (s, BTA8)-IGHJ1 (AY158087) (7.04%). Beside the dominant combinations, 43 were identified solely in one sequence, whereby six were identified in A, 13 in GS, 12 in GBP, and 12 in HF. Again, seven variations rearranging *IGHJ6* were identified.

Fifteen out of the 119 IGHV-IGHD-IGHJ combinations were identified in all four cattle breeds investigated. For A we found 58 different rearrangements, whereas in GBP 65 different recombinations were observed. German Simmental had 57 combinations and HF had 62 variations. Within A (167 rearrangements), the combination IGHV1S39-IGHDQ52 (s, BTA8)-IGHJ1 (AY158087) was the most frequently used and was seen in 11.38% of the time. Chi square calculations showed significant differences in the usage frequencies of the recombinations for breed A (P = 0.0108). In animals of HF (159 rearranged sample sequences), the dominant rearrangement was IGHV1S39-IGHDQ52 (s, BTA8)-IGHJ1 (AY158087) (8.81%). The Chi square test did not show significant usage differences (P = 0.0546) for the different rearranged gene segments in this breed. High significant differences were seen for the recombined IGHV-IGHD-IGHJ in the breed GS (P = 0.0002), while in GBP no significant differences were observed (P = 0.9585). In GS animals rearranged IGHV36/29(F)-IGHDQ52 (s, BTA8)-IGHJ1 (AY158087) (11.45%) was observed predominantly (131 rearranged sequences). The rearrangement IGHV1S40-IGHDQ52 (s, BTA8)-IGHJ1 (AY158087) (9.29%) was preferred in GBP (140 rearranged sequences).

#### Recombination of IGHV, IGHD, and IGHJ with different length of CDR3H

Within the three groups of length of CDR3H we identified different preferably expressed recombinations of *IGHV*, *IGHD*, and *IGHJ*. Very high significant differences regarding identified rearrangements within these groups were calculated (procedure 1: among the breeds, GS, A, HF: P<0.0001; GBP: P = 0.0016; procedure 2: among the breeds, GS, A, HF, GBP: P<0.0001; procedure 3: among the breeds, A, GBP: P<0.0001; GS: P = 0.0003; HF: P = 0.0085).

Using changed penalties for gap opening and gap extension and a new scoring matrix (procedure 3) rearrangements of *IGHV3/33* and *IGHJ1* (AY158087) together with *IGHD1* (s, BTA7), *IGHD2* (as, [40]), *IGHDQ52* (s, BTA8), and *IGHDS10* [22] dominated (0.5–0.84%) in sequences with group 1 CDR3H if breed was not taken into account. The single breeds showed different major recombinations. In A, IGHV6-IGHD2 (s, BTA7)-IGHJ1 was calculated for 1.8% of the sequences, whereas in GS IGHV3/33-IGHDQ52 (s, BTA8)-IGHJ1 was identified in the same frequencies as IGHV3/33-IGHD2 (as, [40])-IGHJ1, IGHV3/33-IGHD1 (as, [40])-IGHJ1, and IGHV1S35-IGHD8 (s, BTA21)-IGHJ1 (0.76%), in GBP IGHV3/33-IGHDS10 [22]-IGHJ1, IGHV3/33-IGHDS14 [22]-IGHJ1, IGHV3/33-IGHD3 (s, BTA7)-IGHJ1, IGHV3/33-IGHD1 (s, BTA7)-IGHJ1, IGHV1S34-IGHDS10 [22]-IGHJ1, IGHV1S34-IGHDS14 [22]-IGHJ1 and IGHV6-IGHDS10 [22]-IGHJ1 made up 0.71%, and in HF IGHV3/33-IGHDQ52 (s, BTA8)-IGHJ1 was found the most often (2.52%).

In samples of all breeds possessing CDR3Hs of group 2 the rearrangement of IGHV36/29(F)-IGHD8 (s, BTA21)-IGHJ1 was found most often (4.52%). This is congruent with our findings from the cattle breeds GS and GBP (10.69%, 5.00%). In A sequences with rearrangement of IGHV1S39-IGHD5 (s, BTA8)-IGHJ1 were used with the highest frequency (7.19%) whereas in HF IGHV3/33-IGHD8 (s, BTA21)-IGHJ1 and IGHV36/29(F)-IGHD3 (s, BTA7)-IGHJ1 dominated (3.77%).

The recombination of IGHV10/34-IGHD2 (s, [40])-IGHJ1 was identified in 0.84% of all sequences with group 3 CDR3Hs. This combination was also dominant in sequences possessing an exceptionally long CDR3H in HF (1.26%). In A, the combination IGHV10/34-IGHD2 (s, BTA7)-IGHJ1 was preferred (1.2%), whereas in GS, IGHV10/34-IGHDS10 [22]-IGHJ1 was found the most often (2.29%). In GBP IGHV10/34-IGHD8 (as, [21])-IGHJ1 and IGHV10/34-IGHD7 (as, [21])-IGHJ1 were identified (0.71%).

If only new values for gap opening and gap extension were applied (procedure 2), rearrangements of *IGHV3/33* and *IGHJ1* (AY158087) together with *IGHD1* (as, [40]) dominated (1.01%) in sequences with a very short CDR3H if the breed was not taken into account. The single breeds showed different major recombinations. In A, IGHV6-IGHD1 (s, BTA7)-IGHJ1 was calculated for 2.4% of the sequences, whereas in GS IGHV3/33-IGHDQ52 (s, BTA8)-IGHJ1 was identified in the same frequencies as IGHV3/33-IGHD4 (as, [21])-IGHJ1, IGHV3/33-IGHD1 (s, BTA7)-IGHJ1, and IGHV1S35-IGHD1 (s, BTA7)-IGHJ1 (0.76%), in GBP IGHV3/33-IGHD1 (as, [40])-IGHJ1 made up 1.43%, whereas in HF IGHV3/33-IGHD1 (as, [40])-IGHJ1 and IGHV3/33-IGHD4 (s, BTA8)-IGHJ1were found the most often (1.26%).

In samples of all breeds possessing group 2 CDR3Hs, the rearrangement of IGHV1S39-IGHDQ52 (s, BTA8)-IGHJ1 was found the most often (7.04%). This is congruent with the findings in the cattle breeds A and HF (11.38%, 8.81%). In GS sequences showing the rearrangement IGHV36/29(F)-IGHDQ52 (s, BTA8)-IGHJ1 were used with the highest frequency (11.45%) whereas in GBP IGHV1S40-IGHDQ52 (s, BTA8)-IGHJ1 dominated (9.29%).

The recombination of IGHV10/34-IGHD8 (s, BTA21)-IGHJ1 was identified in 1.17% of all sequences with group 3 CDR3Hs. This combination was also dominant in sequences possessing exceptionally long CDR3H in A (2.4%) and GBP (0.71%). In GBP IGHV10/34-IGHDS10-IGHJ1 was also found at this frequency. In GS, the combinations IGHV10/34-IGHD1 (s, BTA7)-IGHJ1 and IGHV10/34-IGHD4 (s, BTA8)-IGHJ1 were preferred (2.29%), whereas in HF, IGHV10/34-IGHD3 (s, BTA7)-IGHJ1 was found most often (1.26%).

Using default values (procedure 1) rearrangements of *IGHV3/33* and *IGHJ1* (AY158087) together with *IGHDQ52* (s, BTA8) as well as IGHV6-IGHD1 (s, BTA7) dominated (1.51 and 0.5%) in sequences with a short CDR3H if the breed was not taken into account. But again the single breeds showed different major recombinations. In A, IGHV3/33-IGHDQ52 (s, BTA8)-IGHJ1 and IGHV6-IGHD1 (s, BTA7)-IGHJ1 were found for 1.8% of the sequences, whereas in GS IGHV1S35-IGHD4 (s, BTA8)-IGHJ1 was identified at the same frequency as IGHV3/33-IGHD2 (s, BTA8)-IGHJ1, IGHV3/33-IGHD1 (as, [40])-IGHJ1, and IGHV3/33-IGHD8 (as, [21])-IGHJ1 (0.76%), in GBP IGHV3/33-IGHD1 (s, BTA7)-IGHJ1, IGHV3/33-IGHD2 (as, [40])-IGHJ1, IGHV3/33-IGHD5 (s, BTA8)-IGHJ1, IGHV3/33-IGHDQ52 (s, BTA8)-IGHJ1, IGHV1S34-IGHD5 (s, BTA8)-IGHJ1, IGHV1S34-IGHD5 (s, NW_001503306.)-IGHJ1 and IGHV6-IGHD5 (s, BTA8)-IGHJ1 made up 0.71%, and in HF IGHV3/33-IGHDQ52 (s, BTA8)-IGHJ1 was found most often (3.14%).

In samples of all breeds possessing CDR3Hs of group 2 the rearrangement of IGHV36/29(F)-IGHD8 (s, BTA21)-IGHJ1 was found most often (3.52%). This is congruent with the findings in the cattle breed GS (9.16%). In HF sequences showing the rearrangements IGHV1S39-IGHD8 (s, BTA21)-IGHJ1 and IGHV1S40-IGHD5 (s, BTA8)-IGHJ1 were used with the highest frequency (4.4%) whereas in A IGHV1S39-IGHD3 (s, BTA7)-IGHJ1 dominated (5.99%) as well as IGHV1S40-IGHD8 (s, BTA21)-IGHJ1, IGHV36/29(F)-IGHD5 (s, NW_001503306)-IGHJ1, and IGHV36/29(F)-IGHD8 (s, BTA21)-IGHJ1 in the cattle breed GBP (3.57%).

The recombination of IGHV10/34-IGHD8 (s, BTA21)-IGHJ1 was identified in 1.01% of all sequences with exceptionally long CDR3Hs. In sequences possessing those group 3 CDR3H in HF the combinations IGHV10/34-IGHD4 (s, BTA8)-IGHJ1 and IGHV10/34-IGHD8 (s, BTA21)-IGHJ1 (1.26%) dominated. In A, the combination IGHV10/34-IGHD4 (s, BTA8)-IGHJ1 was preferred (1.2%), whereas in GS, IGHV10/34-IGHD2 (s, BTA7)-IGHJ1 and IGHV10/34-IGHD8 (s, BTA21)-IGHJ1 were found most often (1.53%), and in GBP only the two recombinations IGHV10/34-IGHD8 (s, BTA21)-IGHJ1 and IGHV10/34-IGHD4 (as, [21])-IGHJ1 were identified (0.71%).

### Variability based on amino acid substitutions

**Fig 7:**
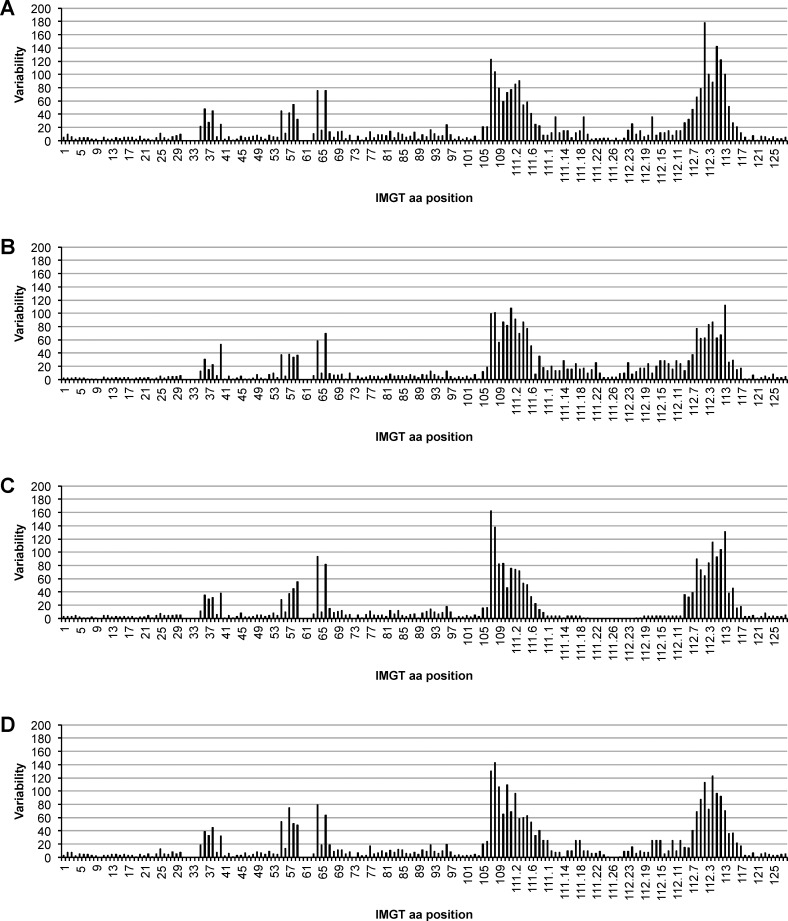
Variability plots of the heavy chain variable regions in the cattle breeds Aubrac, German Simmental, Holstein Friesian, and German Black Pied. The complete variable region is displayed on the horizontal axis. Positions are numbered in accordance to the IMGT numbering system. Within the FR1-4H, little variability is discernable whereas in CDR1-3H increase in variability is observed. (A) Aubrac, (B) German Simmental, (C) German Black Pied, (D) Holstein Friesian

We counted the amino acid substitutions at each position to calculate variability as described by Wu and Kabat [41]. The results are shown in the variability plots for each breed separately in Fig 7A–7D. The amino acid positions were numbered in accordance to the IMGT numbering systems [42]. Therefore, FRHs and CDRHs are defined by the following amino acid positions: FR1H: 1…26, CDR1H: 27…38, FR2H: 39…55, CDR2H: 56…65, FR3H: 66…104, CDR3H: 105…117, FR4H: 118…128. In cattle there are no amino acids assigned to positions 10, 31–34, 60–62, and 73.

Within the FR1-4H little variability is discernable whereas in CDR1-3H an increase in variability is observed as expected. A maximum of variability in FRHs is calculated for position 96 in FR3H. Variability at this position is 23.7 in A, in HF 19.0, whereas in GS and GBP variability is 12.7 and 17.5, respectively. In CDRHs, variability increases from CDR1H to CDR3H in all breeds. In CDR1H breed A showed the highest variability. In CDR2H variability varies between 58 in GS and 93 in GBP. GBP also showed highest variability in CDR3H. The lowest variability within CDR3H was found in GS. Both HF and A showed moderate variability of CDR2H and CDR3H in comparison to GBP and GS. Overall, variability was much higher at the transition areas between FRH and CDR than in the middle of the CDRHs.

## Discussion

This study makes a substantial contribution to the analysis and understanding of the development of the transcribed bovine immunoglobulin repertoire. We examined possible gene conversions within the variable region of bovine heavy chains. We investigated the dominantly transcribed IGHV, IGHD, and IGHJ gene segments and their combinatorial diversity using a newly developed bioinformatics framework, which considers the unique specificity of exceptionally long CDR3 group of bovine immunoglobulin heavy chains. During the development of the program, we applied different conditions (procedure 1 to 3) to improve the alignments of the single gene segments. The progress of assigning germline IGHV, IGHD, and IGHJ to sample sequences is also shown in this study. Unlike previous studies, we complemented our investigation with the analysis of breed specific differences in the four different cattle breeds Aubrac, German Simmental, German Black Pied, and Holstein Friesian.

Due to the limited germline sequence divergence recent studies on bovine immunoglobulin genetics focused on antibody diversification strategies and the junctional diversity of the antibody repertoire. Specific diversification strategies were identified such as the generation of exceptionally long CDR3H [17, 26, 43], the insertion of conserved short nucleotide sequences (CSNS) at the IGHV-IGHD junction [24], the use of pseudogene fragments in lambda light chains as well as gene conversions [14], and somatic hypermutations independent of exposure to external antigens during B-cell development [44]. In addition, more germline gene segments were determined over the last few years [17, 18, 22]. Since the current bovine genome assembly is still incomplete, the full germline repertoire remains under active investigation.

Previous analyses of the bovine immunoglobulin repertoire aimed at identifying rearranged germline gene segments applied various software tools for sequence alignments. As these tools are of limited use for detailed analysis of rearranged immunoglobulin genes due to the difficult and error prone manual assembly of different genes, specialized software tools have been developed. The most familiar ones are IMGT/Junction Analysis [27], IMGT/V-QUEST [28, 29], IMTG/HIGHV-QUEST [30], VBASE2 [31], JoinSolver [32], iHMMun-align [33], and IgBLAST [34]. Only IgBLAST enables the analysis of both nucleotide and protein sequences for FR/CDR and allows the user to either apply the numbering system of Kabat or the IMGT system [42, 45]. Matching germline *IGHV*, *IGHD*, and *IGHJ* genes as well as details at rearrangement junctions may be analyzed. Searches against germline gene databases and other databases are possible [34]. Tools other than IgBLAST do not provide simultaneous database searches or the analysis of protein sequences. All of these immunoglobulin sequence analysis tools support organisms such as human, mouse, rat, rabbit and rhesus monkey, but representation of livestock is missing or incomplete. We did not apply these analysis tools, as they do not consider the bovine specific occurrence of exceptionally long CDR3H. Therefore, we developed a new bioinformatics framework to address this specific case.

In contrast to the analysis tools mentioned above, our program not only searches our updated bovine specific immunoglobulin germline gene database but also is able to load other pre-designed databases. Matches are identified on the basis of nucleotides as it is the case for all other tools. For the delineation of FR and CDR, we apply the IMGT nomenclature that is currently recommended and most widely used. In addition, we focused on the adjustment of search parameters for *IGHV* and *IGHJ* and especially for the identification of *IGHD*.

The analysis of Ig heavy chain variable regions in four cattle breeds revealed the usage of 15 different *IGHV* segments, 21 *IGHD* segments, as well as two *IGHJ* segments. *IGHV1S39* was used most frequently followed by *IGHV3/33*. Rarely used IGHV segments were *IGHV1S26*, *IGHV1S32*, *IGHV1S33*, *IGHV1S37*, and *IGHV1S38*. In bovine fetal bone marrow, ileum, and spleen high frequencies of *IGHV3/33* (= *IGHV1S3/S20*) and *IGHV1S39* was observed as well as low frequencies of *IGHV1S38* and *IGHV1S26* [22]. The transcription of *IGHV1S32* and *S37* has not yet been described. Among the 20, 17, and 21 transcribed *IGHD* (regarding procedures 1–3), *IGHDS8*, *IGHDS5*, *IGHDS10*, and *IGHDQ52* (= *IGHDS9*) were preferred in all breeds. *IGHDS1* to *IGHDS8* were also found to be transcribed in antisense direction in the third calculation procedure but in low numbers. Using the first procedure, *IGHDS6* was not identified in antisense direction, and applying the second procedure *IGHDS1*, *IGHDS4*, *IGHDS6*, and *IGHDS8* were shown to be transcribed in antisense orientation. Previous studies also elucidated the transcription of 14 *IGHD*, where the occurrence of *IGHDS5* was the most frequent one and was present in 42% of the sequences analyzed in bovine fetus [22]. The assignment of the FR4H to germline *IGHJ* revealed the transcription of *IGHJ1*, and *IGHJ6* [22] with *IGHJ1* clearly preferred. In the cattle breed A, procedure 3 confirmed IGHV1S39-IGHD5-IGHJ1 as the most common recombination of gene segments which is identical to the most frequent finding in bovine fetus [22]. This recombination belongs to immunoglobulins possessing a CDR3H region of intermediate length. Statistical analyses showed significant different transcription levels of *IGHV*, *IGHD*, and *IGHJ* segments within the breeds.

The usage of pseudogene segments has already been described for animals such as chicken [4, 38]. In bovine lambda light chains, fragments of pseudogenes were also shown to contribute to immunoglobulin diversity in a gene conversion process [14]. In the current analysis, possible gene conversion events were identified by the assignments of parental germline *IGHV* to separate FR1-3H and CDR1-3H. In addition to the *IGHV* identified for the complete variable region based exclusively on FR1-3H, several pseudogenes were assigned as possible originating germline *IGHV* in the separation analysis. We can exclude allelic variations due to their assignment to different boVH families. For instance, the pseudogenes *IGHV4Ψ/32Ψ*, *IGHV9Ψ/35Ψ*, and *IGHV18Ψ/30Ψ* belong to the boVH2 family [17], but seem to contribute to gene conversion events by nucleotide substitutions. In particular, *IGHV4Ψ/32Ψ*, which was mentioned in the example above meets the criteria for gene conversion such as the location upstream of the rearranged segment *IGHV6* (boVH1) [4, 13] and clusters of nucleotide changes [14]. Further, the flanking homology of the conversion region supports the genetic exchange [13] and the separation from *IGHV6* by more than 18 kb on the genome allows looping during rearrangement [17]. In comparison, in chicken the nearest pseudogene is separated by 7 kb [4]. It should be noted that it is difficult to consider the order of gene segments to evaluate the plausibility of other gene conversions due to the incomplete annotation of the bovine genome [17, 18]. Finally, our data indicate an exchange between the two bovine VH families which obviously is rare and which might be an influence of breed or method of analysis when compared to previous results [18].

The length distribution of CDR3H consists of short CDR3H (group 1), intermediate length CDR3H (group 2), and exceptionally long CDR3H (group 3) in all four cattle breeds. In the breed GS the highest percentage of group 3 CDR3H was calculated. The longest CDR3H with 65 aa were found in GS and HF sequences. The longest ever detected CDR3H in cattle was 67 aa long using IMGT numbering [17]. In contrast, four amino acids made up the shortest CDR3H in A animals, GS animals, and HF animals. The maximum length of group 2 CDR3H was 22 aa.

The combinatorial diversity of germline *IGHV*, *IGHD*, and *IGHJ*-segments is represented by 162 different rearrangements that were expressed with significant differences (procedure 3). In comparison, 147 and 119 different recombinations of IGHV-IGHD-IGHJ were identified using calculation procedures 1 and 2, respectively. In the four breeds, different rearrangements were found. In detail, 91 different combinations occurred in A (procedure 1: 81, and 2: 58), 74 in animals of GBP (procedure 1: 74, and 2: 65), 72 in GS animals (procedure 1: 74, and 2: 57), and 85 in the breed HF (procedure 1: 80, and 2: 62). Most of these combinations were observed in less than ten sequences but seven occurred in up to 21 sequences in all four cattle breeds examined (procedure 1: up to 21, and 2: up to 42).

In sequences belonging to group 1 CDR3H, combinations of *IGHV3/33* and *IGHJ1* (AY158087) together with *IGHDQ52* (s, BTA8) dominated over all breeds using procedure 3. As *IGHDQ52* is the shortest *IGHD* segment possessing only four amino acids, these results explain best the origin of short CDR3H. Group 3 CDR3H mostly exhibited IGHV10/34-IGHD2 (s, [40])-IGHJ1 (procedure 3), or IGHV10/34-IGHD8 (s, BTA21)-IGHJ1 (procedure 1 and 2) in all breeds. Only results from procedure 3 identified biological meaningful combinations of germline IGHV, IGHD, and IGHJ as it gave the best explanation for the origin of group 3 CDR3H. IGHD2 is the longest IGHD segment identified so far. Further, *IGHV10/34*, which is identical to *IGHV1S1* and *IGHV1S15*, was found to contribute solely to those exceptional lengths [17, 18, 22]. Crystallization of two bovine IgG with exceptionally long CDR3H revealed that the “ThrThrValHisGln” terminal motif of IGHV10/34, that initiates an ascending β strand in the folded antibody enables the formation of the “stalk and knob” structure in addition to inserted conserved short nucleotide sequences (CSNS) [22, 26]. Furthermore, in sequences of group 2 a higher number of recombinations were observed than in sequences of group 1 and 3. As group 3 CDR3H regions are unique in cattle, the few preferably rearranged gene segments within this group may indicate specialized antibodies.

Variability plots indicated quite similar features within the variable region in all breeds. Nevertheless, amino acid residues at each position vary between the samples and the breeds and within the regions. In FR1-4H little variability was calculated, whereas the variability increased from CDR1H to CDR3H, which was described already as concentrated areas of diversity in equine heavy and light chain CDRs [46, 47]. The breed A possesses the highest amino acid variations in CDR1H and HF in CDR2H while GS exhibited lowest variability in these two CDRHs. Further, transition areas between FRHs and CDRHs had a higher variability than the middle of CDRHs. Position 96 in FR3H shows the highest variability within the FRHs. This residue is located on the outer surface of the variable region of the immunoglobulin molecule [26] within the area where the constant region is connected to the variable region. The high variability at this position may indicate an influence on the position of variable and constant region and their sterical orientation, which may affect light chain pairing as heavy chains possessing group 3 CDR3H are connected to a special type of lambda light chains [26, 48].

Further analysis revealed that no amino acids were assigned to the IMGT amino acid positions 10, 31–34, 60–62, and 73 in cattle. This means, that one amino acid position within FR1H, four positions in CDR1H, three positions in CDR2H, as well as one position in FR3H were not filled. Consequently, in cattle 8 out of 12 amino acid positions within CDR1H are covered. In CDR2H, 10 positions are available and 7 are covered. Compared to FR-IMGT and CDR-IMGT lengths of functional and ORF *IGHV*-genes of human *IGHV*, mouse *IGHV*, rat *IGHV*, arabian camel *IGHV*, sheep *IGHV*, and pig *IGHV* the missing amino acids within FR1H and FR3H are conserved in all animals mentioned [49, 50]. Averaged eight to ten amino acids were positioned in CDR1H whereas in CDR2H six to ten amino acids were placed by the IMGT numbering system in human, mouse, rat, camel, sheep, and pig *IGHV*. Therefore, the positions of missing amino acids are congruent with other species.

In the breed A, the highest number of recombinations and variability were observed when compared to the other breeds investigated. GS possessed the lowest number of recombinations and showed less variability except in the middle of the CDR3H region. This finding indicates the contribution of insertions and deletions to diversity in case of few rearrangements [25]. It should be noted that A and GS were kept under the same management in a mixed herd. GBP and HF were kept at different farms. The breeds kept in different areas were consequently exposed to different antigens. Thus, the individual number of rearrangements per breed and differences in variability additionally indicate a specialized immune response as animals on one farm are challenged with the same environment.

The application of the newly developed bioinformatics framework led to important new results. Our analyses demonstrated that the bovine heavy chain diversity is not restricted to the use of a limited number of germline genes although there are preferred rearrangements within the three groups of CDR3H lengths. We also found strong evidence for gene conversion using pseudogenes. Despite current advances in the understanding of bovine immunoglobulin diversification, future investigations of the germline repertoire are necessary.

## Material and Methods

### Detailed analyses of immunoglobulin sequences using a newly developed bioinformatics framework

For sequence analysis, we developed a new bioinformatics framework using MUSCLE [35, 36] for the initial fast but accurate multiple nucleotide sequence alignment and following ClustalW [37] for calculating the sequence distances after deduction of the amino acid residues. Both programs are available as stand-alone algorithms and were implemented into our program. The immunogenetics nomenclature (IMGT) was used to assign framework regions 1–4 (FR1-4H) and the complementarity determining regions 1–3 [42].

Therefore, germline nucleotide sequences were imported in FASTA-format (*IGHV*, *IGHD*, *IGHJ* [17, 18, 22]). Using the functional *IGHV*, the nucleotide sequences were translated into amino acids to number the codons of the functional germline *IGHV* gene segments according to the IMGT system from FR1H to FR3H. This required first the identification of the conserved and preassigned positions of Cys23, Trp41, Leu89, and Cys104 defined by Lefranc et al. [42]. Following, the nucleotide sequences of germline pseudo *IGHV* gene segments were aligned separately using MUSCLE [35, 36] to obtain the putative open reading frame. The previously defined positions of the codons using the functional genes were transferred onto the pseudogenes. Insertions as well as deletions of nucleotides within the pseudo gene segment sequence were discarded. The last 33 nucleotides of germline *IGHJ*s were then used to define the FR4H. The region between FR3H and FR4H is defined as CDR3H. This region was later used to align the sample CDR3H to germline *IGHD* segments. Defining FRHs and CDRHs allowed, beside the analyses of the complete transcribed genes, the alignment of *IGHV*, *IGHD* and *IGHJ* using different parameters to improve the biological significance as well as the analyses of the single functionally divergent regions to determine putative gene conversion events in those regions. All functional and pseudo gene germline segments are referred as reference sequences.

Framework regions and CDRs of transcribed sequences (designated as sample sequences) were aligned pairwise to the isolated reference sequences after isolation from the first IgG constant region. Following, minimal divergence was used to identify the most similar sample sequence and reference sequence pairing.

For nucleotide alignments of *IGHV* and *IGHJ*, default values of MUSCLE were used. We tested three different procedures to assign germline and sample *IGHD*s to improve biological significance. In procedure 1, we applied default values of MUSCLE [35, 36], in procedure 2 we changed the penalties for gap opening to -4 and for gap extention to -0.3 [22], and in procedure 3 we additionally incorporated a new scoring matrix with match = 2, transversion = -1, and transition = 1 to evaluate transversion and transition mutations, whereby the IUB (international union of biochemistry) code for single and wobble bases was used.

**Fig 8:**
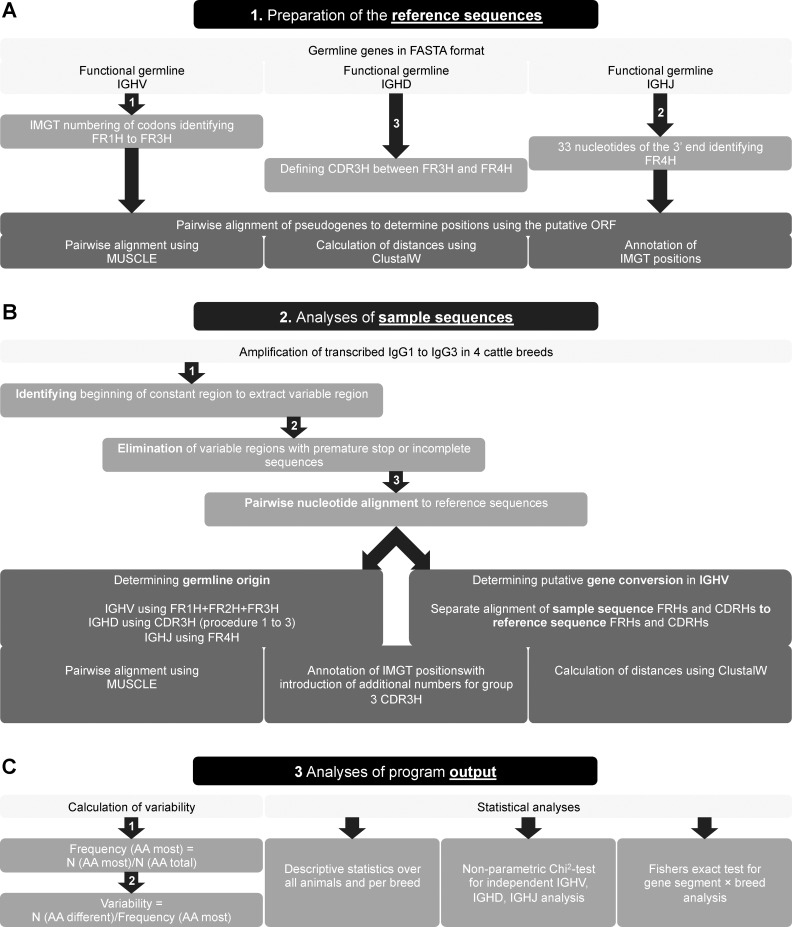
Graphical presentation of workflow of the developed bioinformatics framework used for analyzing bovine heavy chain IgG. (A) At first the reference sequences were prepared. Using the functional segments the pseudogenes were aligned. (B) The bioinformatics framework analyzed the germline origin of bovine immunoglobulin heavy chain variable segments (IGHV), diversity segments (IGHD), and joining segments (IGHJ). One approach used the framework regions (FRH) 1 to 3 to determine the closest germline *IGHV*. The second approach analyzed single functional regions FRH1 to 3 and complementarity determining regions (CDRH) 1 and 2 independently to reveal putative gene conversion events. (C) The last step included the calculation of the variability and the statistical analyses.

The three procedures were applied to a set of sample sequences of IgG-derived variable regions from four different cattle breeds. Each nucleotide sequence of our sample sequences was aligned separately to the reference sequences to determine the most similar reference sequence as germline origin. Following, the codons were translated into amino acids. Sample sequences possessing premature Stop codons or not covering the full length of the variable region due to incomplete sequencing were eliminated and were not incorporated in further analyses. The remaining sample sequences were annotated in accordance to the IMGT nomenclature. For exceptional long CDR3H no positions are defined in the IMGT system, therefore positions had to be added as required and designated as 111.1-111.x and 112.y-112.1 in accordance to the IMGT numbering system [42]. To determine the germline origin, only the FRHs were aligned to avoid interference with the highly diversified CDRH [18]. To analyze possible gene conversion events, FR1-3Hs and CDR1-3Hs were extracted and aligned separately to the corresponding regions of the IGHV reference sequences to find the most similar one (Fig 8). The results were presented as an html table.

To display accumulation of amino acid substitutions in distinct segments of the variable region, variability was calculated as described by Wu and Kabat [41]. Thus, the frequency of the most common amino acid at a distinct position was calculated first. The number of the most abundant amino acid at a given position was divided by the number of all amino acids observed at this position. This means, only samples possessing an amino acid residue at this position in accordance to the IMGT nomenclature were considered. Subsequently, the number of different amino acids at the given position was divided by the frequency of the most common amino acid residue to determine variability. The variability results were written into a txt-file, which allows further analyses in statistical software.

For the statistical analyses of the distribution of *IGHV*, *IGHD*, and *IGHJ* segments including putative gene conversions within one breed and among breeds have been compared by applying non-parametric tests. Such test procedures, i.e. the Chi^2^- test for analyzing *IGHV*, *IGHD* and *IGHJ* independently and Fisher's exact test for the gene segment × breed contingency table, are implemented in the software package SAS, Version 9.2.

### Generation of the sample sequence set

#### Breed selection, isolation of PBMCs and cDNA synthesis

For the analysis of the transcription of IgG heavy chain genes, the four cattle breeds German Black Pied (GBP), German Simmental (GS), Holstein-Friesian (HF), and Aubrac (A) were chosen. The animals selected from the herd of breed A were composed of seven French and three German animals, whereas the sample of breed GS included one Austrian bull. German Black Pied and A represent small populations and have local importance, whereas HF and GS are commonly used in global commercial milk and meat production. German Simmental and A are kept on the same farm under same management conditions and in a mixed herd.

Blood samples were collected from ten randomly chosen animals per breed during routine blood sampling for mandatory examinations in disease control. Peripheral blood mononuclear cells (PBMCs) were isolated using Ficoll gradients (GE Healthcare Europe GmbH, Germany) according to the manufacturer’s protocol. Cells were stained with trypan blue and viable cells were counted. Total RNA was isolated from 1x10^7^ cells using the RNeasy^®^ Mini Kit (Qiagen, Germany). The first-strand cDNA was synthesized using pd(N)_6_ primers from 3 μg of total RNA in a total volume of 20 μl (SuperScript™III First-Strand Synthesis SuperMix, Life Technologies GmbH, Germany).

#### Ethical statement

B-lymphocytes were isolated from already existing blood samples obtained from the tail vein by the herd veterinarian. Blood sampling of cattle is mandatory under German federal and state laws.

#### Amplification of immunoglobulin heavy-chain isotype restricted variable regions

To amplify the variable regions restricted to γ1–3 isotype heavy chains, a PCR was performed with primers binding within the leader region and the 3’UTR (bIgG_leader: ATG AAC CCA CTG TGG ACC; bIgG_3’UTR: CAG GAG GAA TGC ACA CAG). The primers were based on database sequence information and assigned to accession number X62916. The primer boIgG_leader anneals to position 22–39, and the primer boIgG_3’UTR to position 1518–1535. To monitor the integrity and purity of the cDNA, 527 bp of the bovine GAPDH (Glycerinaldehyde 3-phosphate dehydrogenase) were amplified as a positive control. A no- template control served as a negative control for the PCR. The total reaction volume of 50 μl included 0.67 μl of cDNA, 200 μM dNTPs (Bioline, Germany), 5 μl of 10x PCR buffer (75 mM Tris-HCl, pH 9.0; 2 mM MgCl_2_; 50 mM KCl; 20 mM (NH_4_)_2_SO_4_), 0.4 μM of each primer, and 2 units of DNA polymerase (Biotools, Spain). PCR was performed under cycling conditions of 95^°^C for 5 min, followed by 35 cycles of 95^°^C for 1 min, 59.4°C for 1 min, 72^°^C for 2 min, and terminated with elongation at 72°C for 10 min. Length and purity of the PCR products were evaluated by means of electrophoresis on 1% agarose gels.

#### Cloning and sequencing of the PCR products

The PCR products were purified and concentrated using the MiniElute Gel Extraction Kit (Qiagen, Germany) in accordance to the manufacturer’s protocol except QX1 buffer replaced QG buffer. Samples were eluted with 13 μl EB buffer (10 mM Tris-HCl, pH 8.5) and were stored at 4°C. Purified products were cloned into the pCR^®^ 2.1-TOPO^®^ 3.9 kb TA vector (Invitrogen^TM^, Karlsruhe, Germany) and transformed into chemically competent One Shot TOP10 *E*. *coli* cells (Invitrogen^TM^, Karlsruhe, Germany). Transformants were plated on LB agar containing 0.3 mM ampicillin, 40 μl 2.44 μM X-gal (5-bromo-4-chloro-3-indolyl-beta-D-galactopyranoside), and 40 μl 1 M IPTG (Isopropyl β-D-1-thiogalactopyranoside) for blue-white selection. After incubation at 37°C, overnight cultures of randomly selected white transformants were grown in a 5 ml LB-ampicillin broth. Plasmids were isolated using the MiniPrep Kit (Qiagen, Germany). In order to assess the insert size, plasmid DNA was cleaved with *Eco*RI (New England Biolabs, Germany) or a colony PCR was performed. Therefore a 25 μl mixture containing 2 μl cell culture, 0.4 μM of vector specific primers M13 (-20) Forward and M13 Reverse (Invitrogen, Germany), respectively, and one PCR-bead (GE Healthcare Europe GmbH, Germany) were used in a hot start PCR at 95°C for 5 min, denaturation at 95°C for 1 min, annealing at 60°C for 1 min, and extension at 72°C for 2 min up to a total of 30 cycles. A final extension at 72°C for 10 min was included after the final cycle before PCR mixtures were cooled down to 4°C. The size of the resulting fragments and of the PCR products was confirmed by agarose gel electrophoresis.

Sixteen clones per animal were sequenced according to the chain-termination method [51]. The M13 (-20) Forward (5’-GTA AAA CGA CGG CCA G-3’) and M13 Reverse (5’-CAG GAA ACA GCT CTG AC-3’, Invitrogen, Germany) vector-specific primers, as well as the gene specific primers boIgG_leader, boIgG_3’UTR, boIgG_CH1_for (5’-GCC TCC ACC ACA GCC CCG AAA G-3’), boIgG_CH3_rev (5’-GAC CTT GCA CTT GAA CTC C-3’) and boIgG_CH1_rev (5’-ACG GTC ACC ATG CTG CTG AG-3’) were used for sequencing.

## Supporting Information

S1 Fig Transcription frequencies of *IGHD* in four cattle breeds using procedure 1. Transcribed IGHD are shown on the horizontal axis, their relative usage frequencies are indicated on the vertical axis. Calculation occurred using the default values for gap opening and gap extention of MUSCLE. Each breed is marked by the following color code: Aubrac: white, German Simmental: light grey, German Black Pied: dark grey, Holstein Friesian: black
(TIFF)

S2 Fig Transcription frequencies of *IGHD* in four cattle breeds using procedure 2. Transcribed IGHD are shown on the horizontal axis, their relative usage frequencies are indicated on the vertical axis. Calculation occurred after changing the default values for gap opening (-4) and gap extention (-0.3) of MUSCLE. Each breed is marked by the following color code: Aubrac: white, German Simmental: light grey, German Black Pied: dark grey, Holstein Friesian: black
(TIFF)

S3 Fig Recombinations of *IGHV*, *IGHD*, and *IGHJ* over all four cattle breeds using procedure 1. In the sequences of all four cattle breeds analyzed 147 different combinations of *IGHV*, *IGHD*, and *IGHJ* were identified. Relative frequencies (%) of the combinations of the 21 transcribed IGHD and the two transcribed IGHJ are shown depending on the rearranged IGHV (n = 15).
(TIFF)

S4 Fig Recombinations of *IGHV*, *IGHD*, and *IGHJ* over all four cattle breeds using procedure 2. In the sequences of all four cattle breeds analyzed 119 different combinations of *IGHV*, *IGHD*, and *IGHJ* were identified. Relative frequencies (%) of the combinations of the 21 transcribed IGHD and the two transcribed IGHJ are shown depending on the rearranged IGHV (n = 15).
(TIFF)

S1 Table Transcription frequencies of *IGHD* in four cattle breeds using procedure 1. (DOCX)

S2 Table Transcription frequencies of *IGHD* in four cattle breeds using procedure 2. (DOCX)

S3 Table Recombinations of *IGHV*, *IGHD*, and *IGHJ* over all four cattle breeds using procedure 3. (DOCX)

S4 Table Recombinations of *IGHV*, *IGHD*, and *IGHJ* over all four cattle breeds using procedure 1. (DOCX)

S5 Table Recombinations of *IGHV*, *IGHD*, and *IGHJ* over all four cattle breeds using procedure 2. (DOCX)
