# Supplementary figures and images for: Development of a Bioinformatics Framework for the Detection of Gene Conversion and the Analysis of Combinatorial Diversity in Immunoglobulin Heavy Chains in Four Cattle Breeds
Source: PLoS One. 2016 Nov 9;11(11):e0164567. doi: 10.1371/journal.pone.0164567 (PMC5102495; doi:10.1371/journal.pone.0164567)

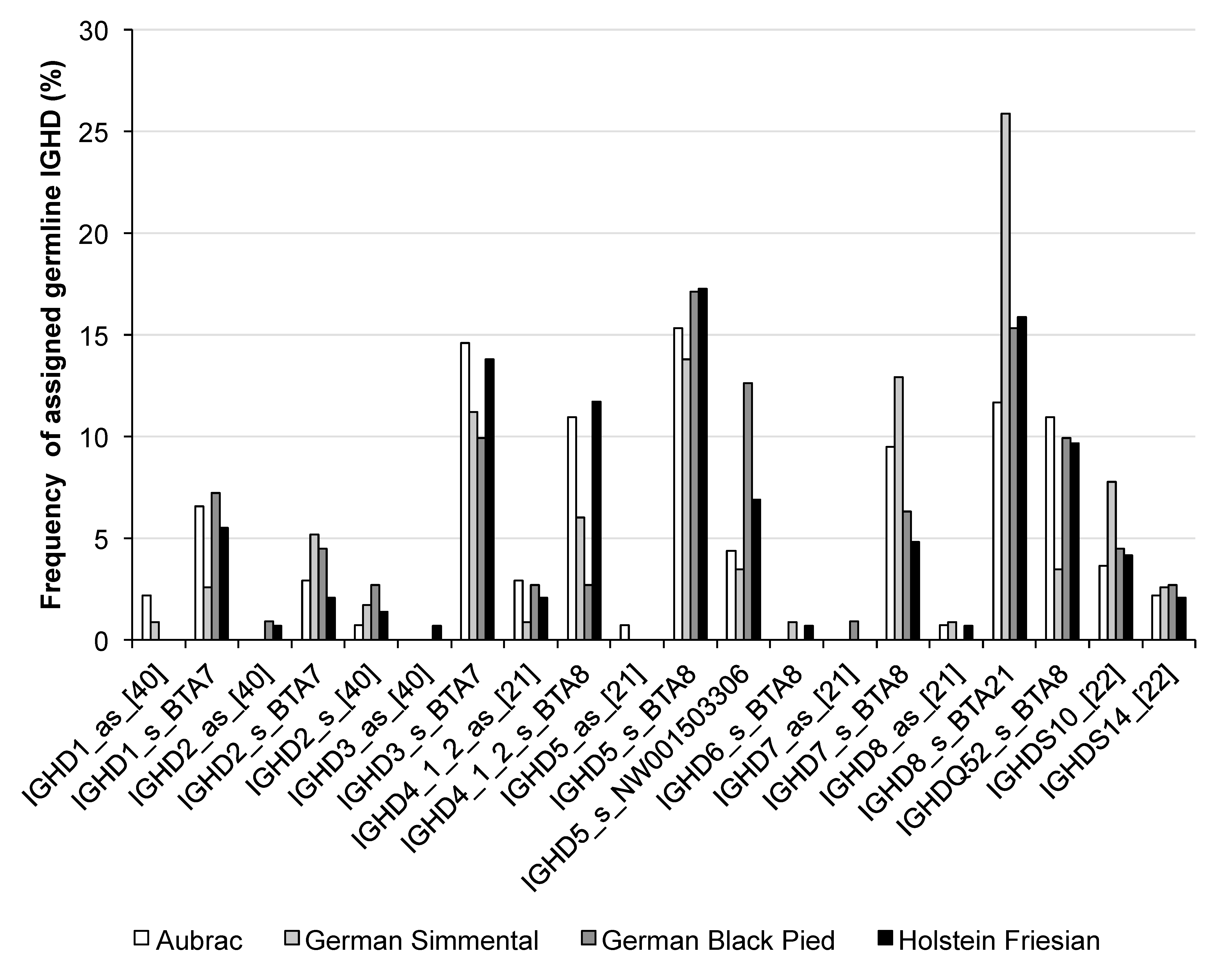

Supplement: S1 Fig — Transcribed IGHD are shown on the horizontal axis, their relative usage frequencies are indicated on the vertical axis. Calculation occurred using the default values for gap opening and gap extention of MUSCLE. Each breed is marked by the following color code: Aubrac: white, German Simmental: light grey, German Black Pied: dark grey, Holstein Friesian: black (TIFF) [file pone.0164567.s001.tiff]

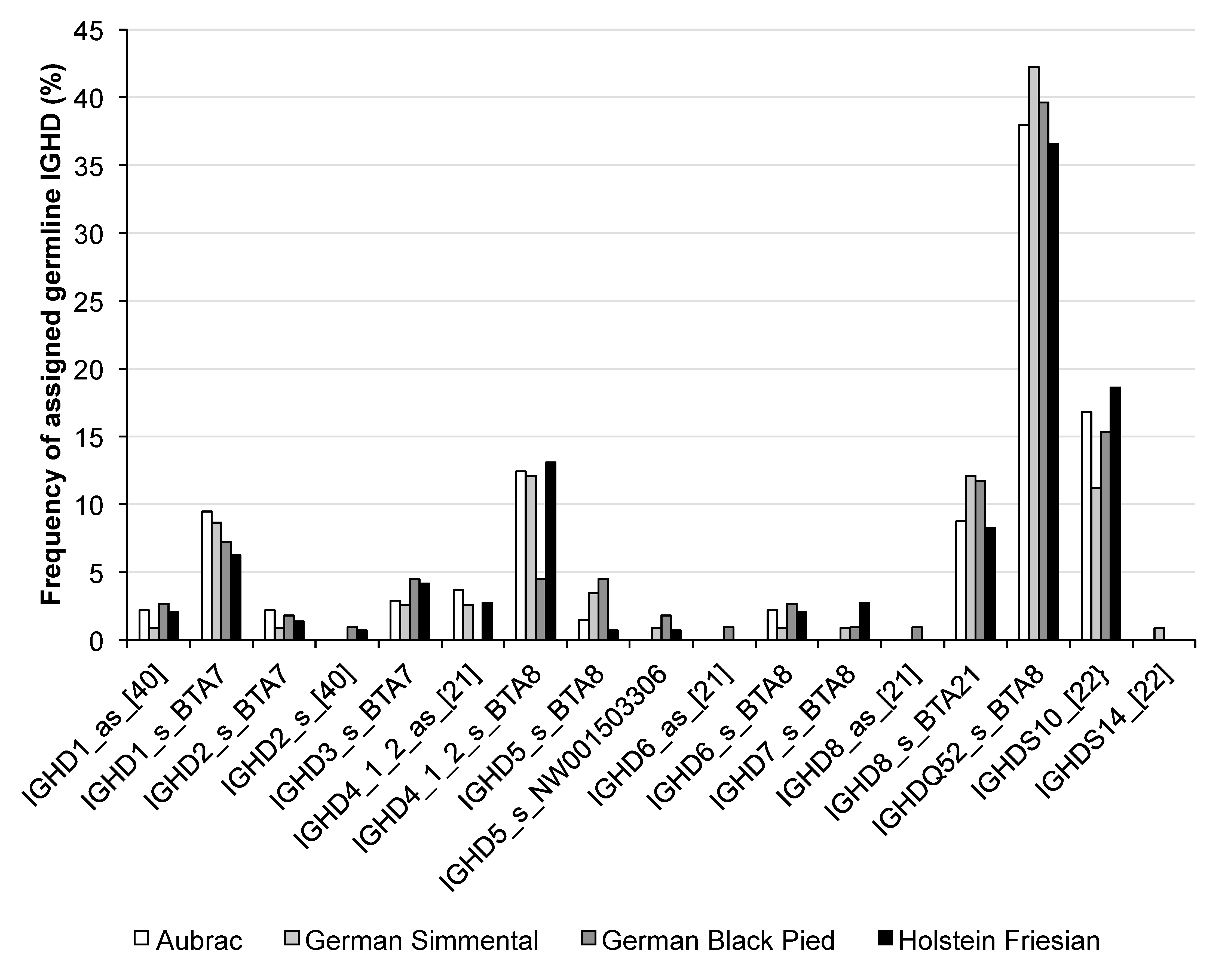

Supplement: S2 Fig — Transcribed IGHD are shown on the horizontal axis, their relative usage frequencies are indicated on the vertical axis. Calculation occurred after changing the default values for gap opening (-4) and gap extention (-0.3) of MUSCLE. Each breed is marked by the following color code: Aubrac: white, German Simmental: light grey, German Black Pied: dark grey, Holstein Friesian: black (TIFF) [file pone.0164567.s002.tiff]

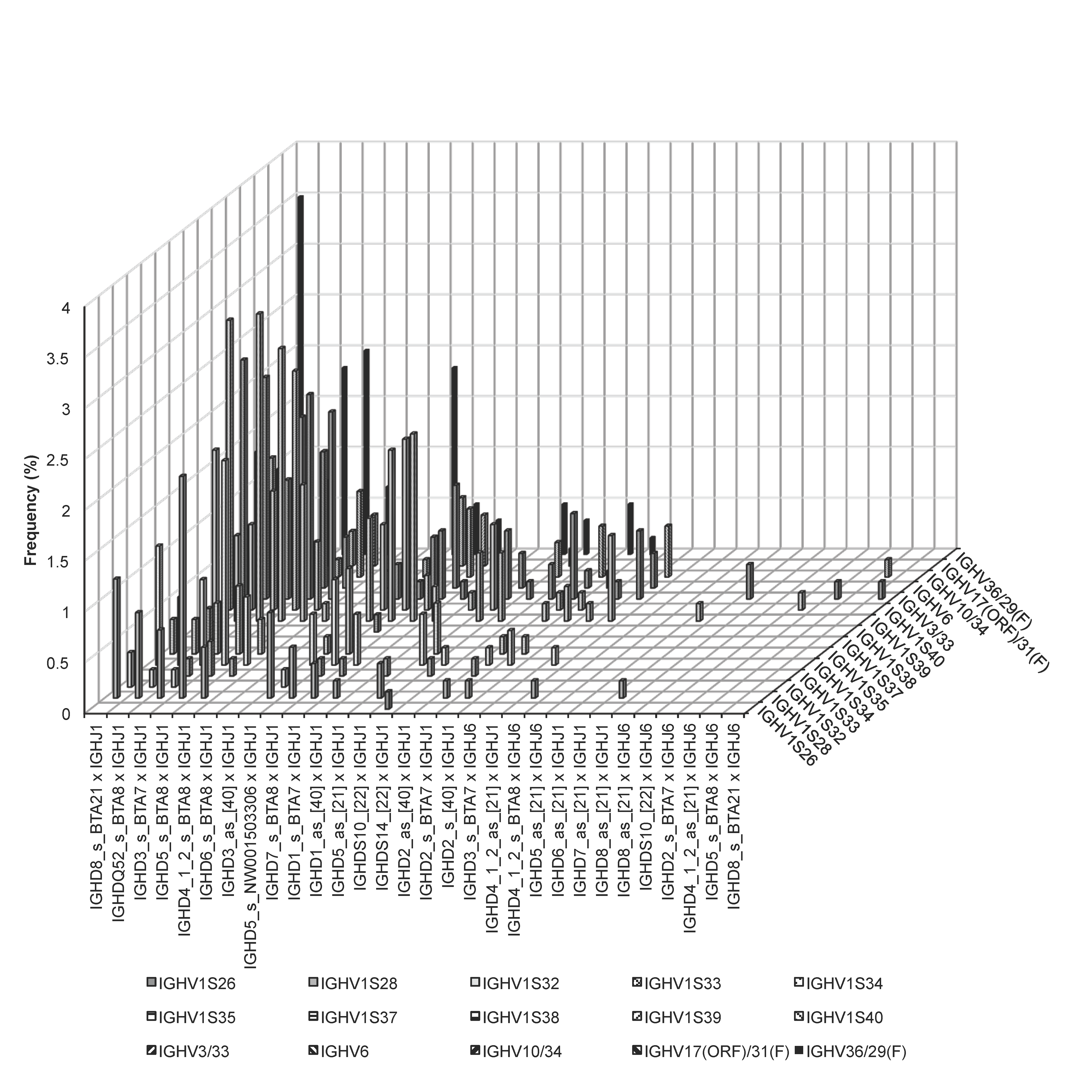

Supplement: S3 Fig — In the sequences of all four cattle breeds analyzed 147 different combinations of IGHV, IGHD, and IGHJ were identified. Relative frequencies (%) of the combinations of the 21 transcribed IGHD and the two transcribed IGHJ are shown depending on the rearranged IGHV (n = 15). (TIFF) [file pone.0164567.s003.tiff]

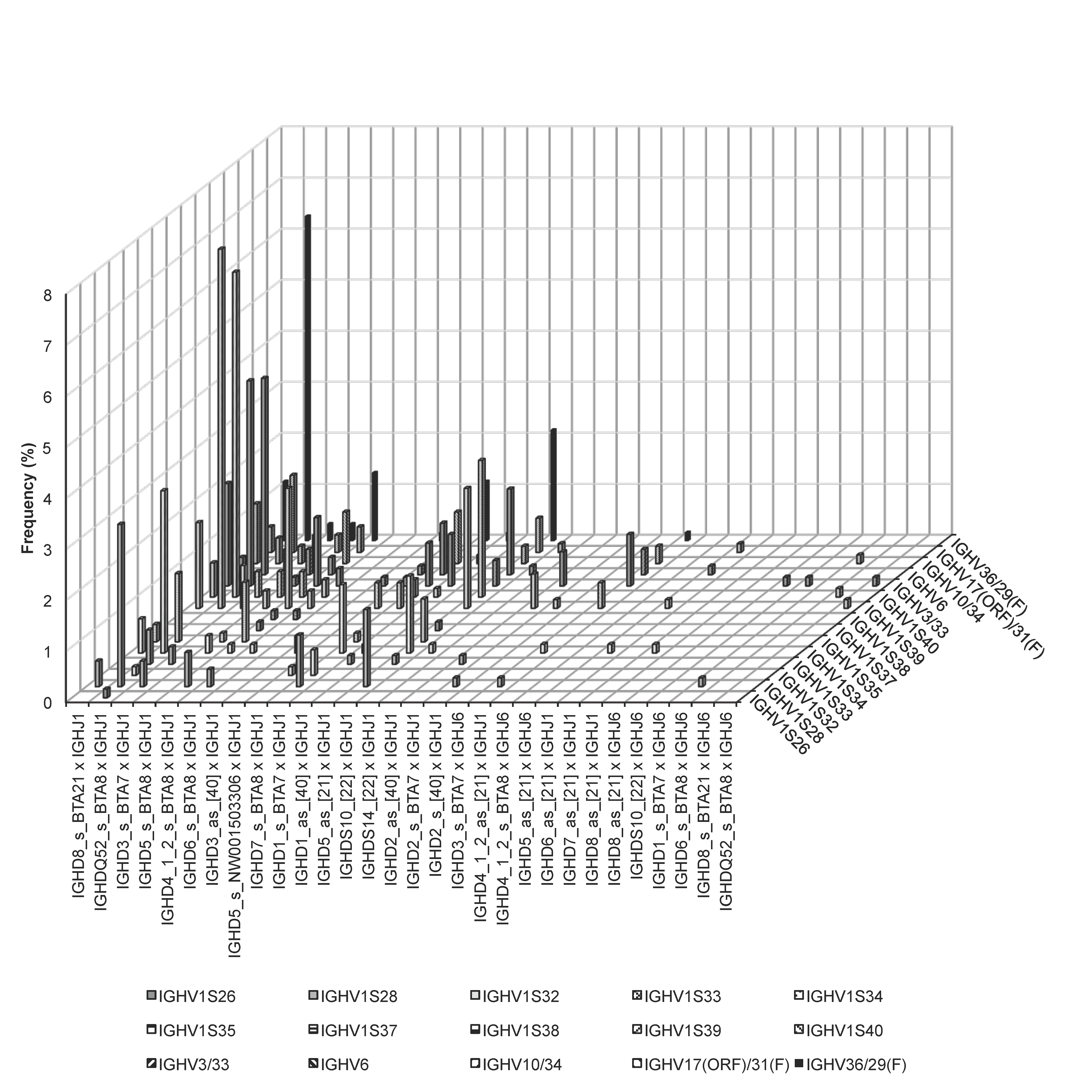

Supplement: S4 Fig — In the sequences of all four cattle breeds analyzed 119 different combinations of IGHV, IGHD, and IGHJ were identified. Relative frequencies (%) of the combinations of the 21 transcribed IGHD and the two transcribed IGHJ are shown depending on the rearranged IGHV (n = 15). (TIFF) [file pone.0164567.s004.tiff]
