# Supplementary material for: Development of a Bioinformatics Framework for the Detection of Gene Conversion and the Analysis of Combinatorial Diversity in Immunoglobulin Heavy Chains in Four Cattle Breeds
Source: PLoS One. 2016 Nov 9;11(11):e0164567. doi: 10.1371/journal.pone.0164567 (PMC5102495; doi:10.1371/journal.pone.0164567)
Supplement: S1 Table — (DOCX) [file pone.0164567.s005.docx]

| **IGHD** | **A**  **(%; n=137)** | **GS**  **(%; n=116)** | **GBP**  **(%; n=111)** | **HF**  **(%; n=145)** |
| --- | --- | --- | --- | --- |
| *IGHD1*_as^1^_[40] | 2.19 | 0.86 | 0.00 | 0.00 |
| *IGHD1*_s^2^_BTA7 | 6.57 | 2.59 | 7.21 | 5.52 |
| *IGHD2*_as_[40] | 0.00 | 0.00 | 0.90 | 0.69 |
| *IGHD2*_s_BTA7 | 2.92 | 5.17 | 4.50 | 2.07 |
| *IGHD2*_s_[40] | 0.73 | 1.72 | 2.70 | 1.38 |
| *IGHD3*_as_[40] | 0.00 | 0.00 | 0.00 | 0.69 |
| *IGHD3*_s_BTA7 | 14.60 | 11.21 | 9.91 | 13.79 |
| *IGHD4*_as_[21] | 2.92 | 0.86 | 2.70 | 2.07 |
| *IGHD4*_s_BTA8 | 10.95 | 6.03 | 2.70 | 11.72 |
| *IGHD5*_as_[21] | 0.73 | 0.00 | 0.00 | 0.00 |
| *IGHD5*_s_BTA8 | 15.33 | 13.79 | 17.12 | 17.24 |
| *IGHD5*_s_NW001503306 | 4.38 | 3.45 | 12.61 | 6.90 |
| *IGHD6*_s_BTA8 | 0.00 | 0.86 | 0.00 | 0.69 |
| *IGHD7*_as_[21] | 0.00 | 0.00 | 0.90 | 0.00 |
| *IGHD7*_s_BTA8 | 9.49 | 12.93 | 6.31 | 4.83 |
| *IGHD8*_as_[21] | 0.73 | 0.86 | 0.00 | 0.69 |
| *IGHD8*_s_BTA21 | 11.68 | 25.86 | 15.32 | 15.86 |
| *IGHDQ52*_s_BTA8 | 10.95 | 3.45 | 9.91 | 9.66 |
| *IGHDS10* [22] | 3.65 | 7.76 | 4.50 | 4.14 |
| *IGHDS14* [22] | 2.19 | 2.59 | 2.70 | 2.07 |

^1^ antisense

^2^ sense
