# Supplementary material for: Development of a Bioinformatics Framework for the Detection of Gene Conversion and the Analysis of Combinatorial Diversity in Immunoglobulin Heavy Chains in Four Cattle Breeds
Source: PLoS One. 2016 Nov 9;11(11):e0164567. doi: 10.1371/journal.pone.0164567 (PMC5102495; doi:10.1371/journal.pone.0164567)
Supplement: S2 Table — (DOCX) [file pone.0164567.s006.docx]

| **IGHD** | **A**  **(%; n=137)** | **GS**  **(%; n=116)** | **GBP**  **(%; n=111)** | **HF**  **(%; n=145)** |
| --- | --- | --- | --- | --- |
| *IGHD1*_as^1^_[40] | 2.19 | 0.86 | 2.70 | 2.07 |
| *IGHD1*_s^2^_BTA7 | 9.49 | 8.62 | 7.21 | 6.21 |
| *IGHD2*_s_BTA7 | 2.19 | 0.86 | 1.80 | 1.38 |
| *IGHD2*_s_[40] | 0.00 | 0.00 | 0.90 | 0.69 |
| *IGHD3*_s_BTA7 | 2.92 | 2.59 | 4.50 | 4.14 |
| *IGHD4*_as_[21] | 3.65 | 2.59 | 0.00 | 2.76 |
| *IGHD4*_s_BTA8 | 12.41 | 12.07 | 4.50 | 13.10 |
| *IGHD5*_s_BTA8 | 1.46 | 3.45 | 4.50 | 0.69 |
| *IGHD5*_s_NW001503306 | 0.00 | 0.86 | 1.80 | 0.69 |
| *IGHD6*_as_[21] | 0.00 | 0.00 | 0.90 | 0.00 |
| *IGHD6*_s_BTA8 | 2.19 | 0.86 | 2.70 | 2.07 |
| *IGHD7*_s_BTA8 | 0.00 | 0.86 | 0.90 | 2.76 |
| *IGHD8*_as_[21] | 0.00 | 0.00 | 0.90 | 0.00 |
| *IGHD8*_s_BTA21 | 8.76 | 12.07 | 11.71 | 8.28 |
| *IGHDQ52*_s_BTA8 | 37.96 | 42.24 | 39.64 | 36.55 |
| *IGHDS10* [22] | 16.79 | 11.21 | 15.32 | 18.62 |
| *IGHDS14* [22] | 0.00 | 0.86 | 0.00 | 0.00 |

^1^ antisense

^2^ sense
