# Supplementary material for: Development of a Bioinformatics Framework for the Detection of Gene Conversion and the Analysis of Combinatorial Diversity in Immunoglobulin Heavy Chains in Four Cattle Breeds
Source: PLoS One. 2016 Nov 9;11(11):e0164567. doi: 10.1371/journal.pone.0164567 (PMC5102495; doi:10.1371/journal.pone.0164567)
Supplement: S3 Table — (DOCX) [file pone.0164567.s007.docx]

| **IGHV - IGHD - IGHJ** | **all**  **(n=597)** | **A**  **(n=167)** | **GBP**  **(n=140)** | **GS**  **(n=131)** | **HF**  **(n=159)** |
| --- | --- | --- | --- | --- | --- |
| IGHV1S26 - IGHD8_s^1^_BTA21 - IGHJ1 | 0.17 | 0.00 | 0.00 | 0.76 | 0.00 |
| IGHV1S28 - IGHD1_as^2^_[40] - IGHJ1 | 0.34 | 0.00 | 1.43 | 0.00 | 0.00 |
| IGHV1S28 - IGHD1_s_BTA7 - IGHJ1 | 0.50 | 1.20 | 0.00 | 0.00 | 0.63 |
| IGHV1S28 - IGHD2_s_[40] - IGHJ1 | 0.17 | 0.00 | 0.00 | 0.76 | 0.00 |
| IGHV1S28 - IGHD3_as_[40] - IGHJ1 | 0.17 | 0.60 | 0.00 | 0.00 | 0.00 |
| IGHV1S28 - IGHD3_s_BTA7 - IGHJ1 | 1.01 | 1.20 | 2.14 | 0.00 | 0.63 |
| IGHV1S28 - IGHD4_s_BTA8 - IGHJ6_[22] | 0.17 | 0.00 | 0.00 | 0.00 | 0.63 |
| IGHV1S28 - IGHD4_s_BTA8 - IGHJ1 | 1.17 | 1.20 | 1.43 | 0.76 | 1.26 |
| IGHV1S28 - IGHD5_s_BTA8 - IGHJ1 | 1.17 | 1.20 | 0.71 | 2.29 | 0.63 |
| IGHV1S28 - IGHD5_s_NW_001503306 - IGHJ1 | 0.34 | 0.60 | 0.00 | 0.76 | 0.00 |
| IGHV1S28 - IGHD6_s_BTA8 - IGHJ1 | 0.17 | 0.00 | 0.71 | 0.00 | 0.00 |
| IGHV1S28 - IGHD7_s_BTA8 - IGHJ1 | 0.84 | 0.00 | 1.43 | 1.53 | 0.63 |
| IGHV1S28 - IGHD8_s_BTA21 - IGHJ1 | 0.34 | 0.00 | 0.00 | 0.76 | 0.63 |
| IGHV1S28 - IGHDQ52_s_BTA8 - IGHJ1 | 0.84 | 0.00 | 0.71 | 0.76 | 1.89 |
| IGHV1S28 - IGHDS10 [22] - IGHJ1 | 0.67 | 0.00 | 0.00 | 0.00 | 2.52 |
| IGHV1S28 - IGHDS14 [22] - IGHJ1 | 0.34 | 0.60 | 0.00 | 0.76 | 0.00 |
| IGHV1S32 - IGHD2_s_[40] - IGHJ1 | 0.17 | 0.00 | 0.00 | 0.76 | 0.00 |
| IGHV1S32 - IGHD8_s_BTA21 - IGHJ1 | 0.34 | 0.00 | 0.00 | 1.53 | 0.00 |
| IGHV1S32 - IGHDS10 [22] - IGHJ1 | 0.17 | 0.00 | 0.00 | 0.00 | 0.63 |
| IGHV1S32 - IGHDS14 [22] - IGHJ1 | 0.17 | 0.00 | 0.00 | 0.00 | 0.63 |
| IGHV1S33 - IGHD3_as_[40] - IGHJ1 | 0.17 | 0.60 | 0.00 | 0.00 | 0.00 |
| IGHV1S33 - IGHD3_s_BTA7 - IGHJ1 | 0.34 | 1.20 | 0.00 | 0.00 | 0.00 |
| IGHV1S33 - IGHD4_s_BTA8 - IGHJ1 | 0.17 | 0.00 | 0.71 | 0.00 | 0.00 |
| IGHV1S33 - IGHD5_s_BTA8 - IGHJ1 | 0.17 | 0.00 | 0.00 | 0.76 | 0.00 |
| IGHV1S33 - IGHD8_s_BTA21 - IGHJ1 | 0.17 | 0.60 | 0.00 | 0.00 | 0.00 |
| IGHV1S33 - IGHDQ52_s_BTA8 - IGHJ1 | 0.34 | 1.20 | 0.00 | 0.00 | 0.00 |
| IGHV1S33 - IGHDS14 [22] - IGHJ1 | 0.17 | 0.00 | 0.00 | 0.76 | 0.00 |
| IGHV1S34 - IGHD1_as_[40] - IGHJ1 | 0.17 | 0.60 | 0.00 | 0.00 | 0.00 |
| IGHV1S34 - IGHD1_s_BTA7 - IGHJ1 | 1.34 | 0.60 | 3.57 | 0.76 | 0.63 |
| IGHV1S34 - IGHD2_s_BTA7 - IGHJ1 | 0.34 | 0.60 | 0.00 | 0.00 | 0.63 |
| IGHV1S34 - IGHD2_s_[40] - IGHJ1 | 0.17 | 0.00 | 0.00 | 0.76 | 0.00 |
| IGHV1S34 - IGHD3_s_BTA7 - IGHJ1 | 0.84 | 0.00 | 2.14 | 0.76 | 0.63 |
| IGHV1S34 - IGHD4_s_BTA8 - IGHJ1 | 0.67 | 0.60 | 1.43 | 0.00 | 0.63 |
| IGHV1S34 - IGHD5_s_BTA8 - IGHJ1 | 1.01 | 0.60 | 1.43 | 0.76 | 1.26 |
| IGHV1S34 - IGHD5_s_NW_001503306 - IGHJ1 | 0.34 | 0.00 | 0.71 | 0.00 | 0.63 |
| IGHV1S34 - IGHD6_s_BTA8 - IGHJ1 | 0.17 | 0.00 | 0.00 | 0.76 | 0.00 |
| IGHV1S34 - IGHD7_s_BTA8 - IGHJ1 | 0.34 | 0.60 | 0.71 | 0.00 | 0.00 |
| IGHV1S34 - IGHD8_s_BTA21 - IGHJ1 | 1.01 | 0.00 | 0.71 | 3.82 | 0.00 |
| IGHV1S34 - IGHDQ52_s_BTA8 - IGHJ1 | 1.51 | 2.40 | 2.14 | 0.76 | 0.63 |
| IGHV1S34 - IGHDS10 [22] - IGHJ1 | 0.34 | 0.00 | 0.71 | 0.00 | 0.63 |
| IGHV1S34 - IGHDS14 [22] - IGHJ1 | 0.34 | 0.00 | 0.00 | 1.53 | 0.00 |
| IGHV1S35 - IGHD1_s_BTA7 - IGHJ1 | 0.17 | 0.00 | 0.00 | 0.00 | 0.63 |
| IGHV1S35 - IGHD2_s_[40] - IGHJ1 | 0.17 | 0.00 | 0.00 | 0.76 | 0.00 |
| IGHV1S35 - IGHD3_s_BTA7 - IGHJ1 | 0.50 | 0.00 | 0.71 | 1.53 | 0.00 |
| IGHV1S35 - IGHD4_as_[21] - IGHJ1 | 0.34 | 0.60 | 0.00 | 0.76 | 0.00 |
| IGHV1S35 - IGHD4_s_BTA8 - IGHJ1 | 0.34 | 0.60 | 0.00 | 0.76 | 0.00 |
| IGHV1S35 - IGHD5_s_BTA8 - IGHJ1 | 0.50 | 0.00 | 0.00 | 2.29 | 0.00 |
| IGHV1S35 - IGHD7_s_BTA8 - IGHJ1 | 1.01 | 0.00 | 0.71 | 3.82 | 0.00 |
| IGHV1S35 - IGHD8_s_BTA21 - IGHJ1 | 0.67 | 1.20 | 0.00 | 1.53 | 0.00 |
| IGHV1S35 - IGHDQ52_s_BTA8 - IGHJ1 | 0.17 | 0.60 | 0.00 | 0.00 | 0.00 |
| IGHV1S35 - IGHDS10 [22] - IGHJ1 | 0.17 | 0.00 | 0.00 | 0.00 | 0.63 |
| IGHV1S37 - IGHD4_s_BTA8 - IGHJ1 | 0.17 | 0.00 | 0.00 | 0.00 | 0.63 |
| IGHV1S37 - IGHDQ52_s_BTA8 - IGHJ1 | 0.17 | 0.00 | 0.00 | 0.00 | 0.63 |
| IGHV1S38 - IGHD3_s_BTA7 - IGHJ1 | 0.17 | 0.00 | 0.00 | 0.76 | 0.00 |
| IGHV1S38 - IGHDQ52_s_BTA8 - IGHJ1 | 0.17 | 0.00 | 0.00 | 0.00 | 0.63 |
| IGHV1S39 - IGHD1_as_[40] - IGHJ1 | 0.34 | 0.60 | 0.00 | 0.00 | 0.63 |
| IGHV1S39 - IGHD1_s_BTA7 - IGHJ1 | 1.01 | 1.20 | 1.43 | 0.00 | 1.26 |
| IGHV1S39 - IGHD2_as_[40] - IGHJ1 | 0.34 | 1.20 | 0.00 | 0.00 | 0.00 |
| IGHV1S39 - IGHD2_s_BTA7 - IGHJ1 | 0.34 | 0.60 | 0.00 | 0.00 | 0.63 |
| IGHV1S39 - IGHD2_s_[40] - IGHJ1 | 0.84 | 0.00 | 1.43 | 0.76 | 1.26 |
| IGHV1S39 - IGHD3_s_BTA7 - IGHJ6_[22] | 0.17 | 0.00 | 0.00 | 0.00 | 0.63 |
| IGHV1S39 - IGHD3_s_BTA7 - IGHJ1 | 1.01 | 1.20 | 1.43 | 0.00 | 1.26 |
| IGHV1S39 - IGHD4_as_[21] - IGHJ1 | 0.50 | 0.60 | 0.00 | 0.76 | 0.63 |
| IGHV1S39 - IGHD4_s_BTA8 - IGHJ1 | 1.68 | 3.59 | 0.71 | 0.00 | 1.89 |
| IGHV1S39 - IGHD5_s_BTA8 - IGHJ1 | 2.85 | 7.78 | 0.71 | 0.00 | 1.89 |
| IGHV1S39 - IGHD5_s_NW_001503306 - IGHJ1 | 1.17 | 1.20 | 2.86 | 0.76 | 0.00 |
| IGHV1S39 - IGHD6_as_[21] - IGHJ1 | 0.34 | 0.00 | 0.00 | 0.00 | 1.26 |
| IGHV1S39 - IGHD6_s_BTA8 - IGHJ1 | 0.34 | 0.00 | 0.00 | 0.00 | 1.26 |
| IGHV1S39 - IGHD7_s_BTA8 - IGHJ1 | 1.68 | 2.40 | 0.71 | 0.76 | 2.52 |
| IGHV1S39 - IGHD8_s_BTA21 - IGHJ1 | 2.18 | 4.79 | 1.43 | 0.00 | 1.89 |
| IGHV1S39 - IGHDQ52_s_BTA8 - IGHJ1 | 1.34 | 2.99 | 0.00 | 0.00 | 1.89 |
| IGHV1S39 - IGHDS10 [22] - IGHJ1 | 1.01 | 1.20 | 2.86 | 0.00 | 0.00 |
| IGHV1S39 - IGHDS14 [22] - IGHJ1 | 0.50 | 1.20 | 0.00 | 0.00 | 0.63 |
| IGHV1S40 - IGHD1_s_BTA7 - IGHJ1 | 0.17 | 0.00 | 0.00 | 0.00 | 0.63 |
| IGHV1S40 - IGHD2_s_BTA7 - IGHJ1 | 0.17 | 0.60 | 0.00 | 0.00 | 0.00 |
| IGHV1S40 - IGHD2_s_[40] - IGHJ1 | 0.17 | 0.00 | 0.00 | 0.76 | 0.00 |
| IGHV1S40 - IGHD3_as_[40] - IGHJ1 | 0.17 | 0.60 | 0.00 | 0.00 | 0.00 |
| IGHV1S40 - IGHD3_s_BTA7 - IGHJ1 | 2.35 | 0.00 | 2.86 | 4.58 | 2.52 |
| IGHV1S40 - IGHD4_as_[21] - IGHJ1 | 0.17 | 0.60 | 0.00 | 0.00 | 0.00 |
| IGHV1S40 - IGHD4_s_BTA8 - IGHJ1 | 0.84 | 0.60 | 1.43 | 0.00 | 1.26 |
| IGHV1S40 - IGHD5_s_BTA8 - IGHJ1 | 3.02 | 2.99 | 3.57 | 4.58 | 1.26 |
| IGHV1S40 - IGHD5_s_NW_001503306 - IGHJ1 | 0.50 | 0.60 | 0.71 | 0.00 | 0.63 |
| IGHV1S40 - IGHD6_as_[21] - IGHJ1 | 0.17 | 0.00 | 0.71 | 0.00 | 0.00 |
| IGHV1S40 - IGHD6_s_BTA8 - IGHJ1 | 0.17 | 0.60 | 0.00 | 0.00 | 0.00 |
| IGHV1S40 - IGHD7_s_BTA8 - IGHJ1 | 0.84 | 0.00 | 1.43 | 1.53 | 0.63 |
| IGHV1S40 - IGHD8_s_BTA21 - IGHJ1 | 1.34 | 1.20 | 2.14 | 0.76 | 1.26 |
| IGHV1S40 - IGHDQ52_s_BTA8 - IGHJ1 | 1.51 | 0.60 | 1.43 | 2.29 | 1.89 |
| IGHV1S40 - IGHDS10 [22] - IGHJ6_[22] | 0.17 | 0.00 | 0.71 | 0.00 | 0.00 |
| IGHV1S40 - IGHDS10 [22] - IGHJ1 | 0.50 | 0.00 | 0.71 | 0.76 | 0.63 |
| IGHV3/33 - IGHD1_as_[40] - IGHJ1 | 0.34 | 0.60 | 0.00 | 0.76 | 0.00 |
| IGHV3/33 - IGHD1_s_BTA7 - IGHJ1 | 1.51 | 0.00 | 2.14 | 0.76 | 3.14 |
| IGHV3/33 - IGHD2_as_[40] - IGHJ1 | 0.50 | 1.20 | 0.00 | 0.76 | 0.00 |
| IGHV3/33 - IGHD2_s_BTA7 - IGHJ1 | 0.50 | 0.60 | 0.00 | 0.76 | 0.63 |
| IGHV3/33 - IGHD2_s_[40] - IGHJ1 | 0.34 | 0.00 | 0.71 | 0.00 | 0.63 |
| IGHV3/33 - IGHD3_as_[40] - IGHJ1 | 0.17 | 0.60 | 0.00 | 0.00 | 0.00 |
| IGHV3/33 - IGHD3_s_BTA7 - IGHJ1 | 1.34 | 1.20 | 2.14 | 0.76 | 1.26 |
| IGHV3/33 - IGHD3_s_BTA7 - IGHJ6_[22] | 0.34 | 0.00 | 0.00 | 0.76 | 0.63 |
| IGHV3/33 - IGHD4_as_[21] - IGHJ1 | 0.50 | 0.00 | 0.71 | 0.00 | 1.26 |
| IGHV3/33 - IGHD4_s_BTA8 - IGHJ1 | 0.50 | 1.20 | 0.71 | 0.00 | 0.00 |
| IGHV3/33 - IGHD5_as_[21] - IGHJ1 | 0.17 | 0.60 | 0.00 | 0.00 | 0.00 |
| IGHV3/33 - IGHD5_s_BTA8 - IGHJ1 | 2.01 | 1.20 | 1.43 | 2.29 | 3.14 |
| IGHV3/33 - IGHD5_s_NW_001503306 - IGHJ1 | 0.50 | 0.60 | 0.00 | 0.00 | 1.26 |
| IGHV3/33 - IGHD7_s_BTA8 - IGHJ1 | 0.50 | 0.60 | 1.43 | 0.00 | 0.00 |
| IGHV3/33 - IGHD8_as_[21] IGHJ6_[22] | 0.17 | 0.60 | 0.00 | 0.00 | 0.00 |
| IGHV3/33 - IGHD8_s_BTA21 - IGHJ1 | 1.68 | 0.60 | 0.71 | 0.76 | 4.40 |
| IGHV3/33 - IGHDQ52_s_BTA8 - IGHJ1 | 1.84 | 0.00 | 0.71 | 3.05 | 3.77 |
| IGHV3/33 - IGHDS10 [22] - IGHJ1 | 1.01 | 2.40 | 0.71 | 0.76 | 0.00 |
| IGHV3/33 - IGHDS14 [22] - IGHJ1 | 0.50 | 0.00 | 1.43 | 0.76 | 0.00 |
| IGHV6 - IGHD1_as_[40] - IGHJ1 | 0.17 | 0.60 | 0.00 | 0.00 | 0.00 |
| IGHV6 - IGHD1_s_BTA7 - IGHJ1 | 0.67 | 0.60 | 1.43 | 0.00 | 0.63 |
| IGHV6 - IGHD2_s_BTA7 - IGHJ1 | 0.50 | 1.80 | 0.00 | 0.00 | 0.00 |
| IGHV6 - IGHD2_s_[40] - IGHJ1 | 0.17 | 0.00 | 0.00 | 0.76 | 0.00 |
| IGHV6 - IGHD3_as_[40] - IGHJ1 | 0.34 | 0.00 | 0.71 | 0.76 | 0.00 |
| IGHV6 - IGHD3_s_BTA7 - IGHJ1 | 0.84 | 0.60 | 1.43 | 0.00 | 1.26 |
| IGHV6 - IGHD4_as_[21] - IGHJ1 | 0.34 | 0.60 | 0.00 | 0.00 | 0.63 |
| IGHV6 - IGHD4_s_BTA8 - IGHJ1 | 1.01 | 1.20 | 0.00 | 0.76 | 1.89 |
| IGHV6 - IGHD5_s_BTA8 - IGHJ1 | 0.84 | 0.60 | 1.43 | 0.00 | 1.26 |
| IGHV6 - IGHD5_s_NW_001503306 - IGHJ1 | 0.50 | 0.60 | 0.71 | 0.76 | 0.00 |
| IGHV6 - IGHD7_s_BTA8 - IGHJ1 | 0.50 | 0.60 | 0.71 | 0.00 | 0.63 |
| IGHV6 - IGHD8_s_BTA21 IGHJ1 | 1.34 | 1.80 | 0.71 | 3.05 | 0.00 |
| IGHV6 - IGHDQ52_s_BTA8 - IGHJ1 | 1.34 | 1.80 | 1.43 | 0.00 | 1.89 |
| IGHV6 - IGHDS10 [22] - IGHJ1 | 0.34 | 0.00 | 0.71 | 0.00 | 0.63 |
| IGHV6 - IGHDS14 [22] - IGHJ1 | 0.34 | 0.00 | 0.00 | 1.53 | 0.00 |
| IGHV10/34 - IGHD2_as_[40] - IGHJ1 | 0.34 | 1.20 | 0.00 | 0.00 | 0.00 |
| IGHV10/34 - IGHD1_s_BTA7 - IGHJ1 | 0.34 | 0.60 | 0.00 | 0.00 | 0.63 |
| IGHV10/34 - IGHD2_s_BTA7 - IGHJ1 | 0.50 | 1.20 | 0.00 | 0.76 | 0.00 |
| IGHV10/34 - IGHD2_s_[40] - IGHJ1 | 1.01 | 0.60 | 0.00 | 2.29 | 1.26 |
| IGHV10/34 - IGHD3_s_BTA7 - IGHJ1 | 0.67 | 0.60 | 0.00 | 0.76 | 1.26 |
| IGHV10/34 - IGHD4_s_BTA8 - IGHJ1 | 0.17 | 0.60 | 0.00 | 0.00 | 0.00 |
| IGHV10/34 - IGHD5_as_[21] - IGHJ6_[22] | 0.17 | 0.00 | 0.00 | 0.00 | 0.63 |
| IGHV10/34 - IGHD5_s_BTA8 - IGHJ1 | 0.17 | 0.00 | 0.00 | 0.00 | 0.63 |
| IGHV10/34 - IGHD5_s_NW_001503306 - IGHJ1 | 0.17 | 0.00 | 0.00 | 0.00 | 0.63 |
| IGHV10/34 - IGHD7_as_[21] - IGHJ1 | 0.17 | 0.00 | 0.71 | 0.00 | 0.00 |
| IGHV10/34 - IGHD7_s_BTA8 - IGHJ1 | 0.17 | 0.00 | 0.00 | 0.76 | 0.00 |
| IGHV10/34 - IGHD8_as_[21] - IGHJ1 | 0.34 | 0.00 | 0.71 | 0.00 | 0.63 |
| IGHV10/34 - IGHD8_s_BTA21 - IGHJ1 | 0.17 | 0.60 | 0.00 | 0.00 | 0.00 |
| IGHV10/34 - IGHDQ52_s_BTA8 - IGHJ1 | 0.17 | 0.60 | 0.00 | 0.00 | 0.00 |
| IGHV10/34 - IGHDS10 [22] - IGHJ1 | 0.50 | 0.00 | 0.00 | 2.29 | 0.00 |
| IGHV17(ORF)/31(F) - IGHD1_s_BTA7 - IGHJ1 | 0.50 | 0.60 | 1.43 | 0.00 | 0.00 |
| IGHV17(ORF)/31(F) - IGHD3_s_BTA7 - IGHJ1 | 1.01 | 1.80 | 0.71 | 1.53 | 0.00 |
| IGHV17(ORF)/31(F) - IGHD4_as_[21] - IGHJ1 | 0.17 | 0.60 | 0.00 | 0.00 | 0.00 |
| IGHV17(ORF)/31(F) - IGHD4_s_BTA8 - IGHJ1 | 0.34 | 0.60 | 0.71 | 0.00 | 0.00 |
| IGHV17(ORF)/31(F) - IGHD5_s_BTA8 - IGHJ1 | 0.50 | 0.00 | 1.43 | 0.00 | 0.63 |
| IGHV17(ORF)/31(F) - IGHD5_s_NW_001503306 - IGHJ1 | 0.17 | 0.00 | 0.71 | 0.00 | 0.00 |
| IGHV17(ORF)/31(F) - IGHD7_s_BTA8 - IGHJ1 | 0.34 | 0.00 | 0.71 | 0.00 | 0.63 |
| IGHV17(ORF)/31(F) - IGHD8_s_BTA21 - IGHJ1 | 0.34 | 0.00 | 0.00 | 1.53 | 0.00 |
| IGHV17(ORF)/31(F) - IGHDQ52_s_BTA8 - IGHJ1 | 0.50 | 1.20 | 0.00 | 0.00 | 0.63 |
| IGHV17(ORF)/31(F) - IGHDS10 [22] - IGHJ1 | 0.34 | 0.60 | 0.71 | 0.00 | 0.00 |
| IGHV36/29(F) - IGHD1_as_[40] - IGHJ1 | 0.17 | 0.60 | 0.00 | 0.00 | 0.00 |
| IGHV36/29(F) - IGHD1_s_BTA7 - IGHJ1 | 0.34 | 0.60 | 0.00 | 0.00 | 0.63 |
| IGHV36/29(F) - IGHD3_as_[40] - IGHJ1 | 0.50 | 0.60 | 0.71 | 0.76 | 0.00 |
| IGHV36/29(F) - IGHD3_s_BTA7 - IGHJ1 | 1.51 | 0.00 | 2.14 | 0.00 | 3.77 |
| IGHV36/29(F) - IGHD4_s_BTA8 - IGHJ1 | 0.84 | 0.00 | 0.00 | 1.53 | 1.89 |
| IGHV36/29(F) - IGHD5_as_[21] - IGHJ1 | 0.17 | 0.00 | 0.00 | 0.76 | 0.00 |
| IGHV36/29(F) - IGHD5_s_BTA8 - IGHJ1 | 1.51 | 1.80 | 2.86 | 0.76 | 0.63 |
| IGHV36/29(F) - IGHD5_s_NW_001503306 - IGHJ1 | 0.50 | 0.00 | 0.71 | 0.00 | 1.26 |
| IGHV36/29(F) - IGHD6_s_BTA8 - IGHJ1 | 0.67 | 0.00 | 1.43 | 0.00 | 1.26 |
| IGHV36/29(F) - IGHD7_s_BTA8 - IGHJ1 | 0.50 | 0.60 | 0.00 | 0.76 | 0.63 |
| IGHV36/29(F) - IGHD8_s_BTA21 - IGHJ1 | 4.52 | 1.20 | 5.00 | 10.69 | 2.52 |
| IGHV36/29(F) - IGHDQ52_s_BTA8 - IGHJ1 | 1.84 | 1.80 | 3.57 | 0.76 | 1.26 |
| IGHV36/29(F) - IGHDS10 [22] - IGHJ1 | 0.17 | 0.00 | 0.00 | 0.76 | 0.00 |
| IGHV36/29(F) - IGHDS14 [22] - IGHJ1 | 0.17 | 0.60 | 0.00 | 0.00 | 0.00 |

^1^ sense

^2^ antisense
